# Antibiotic-free antimicrobial functionalization of PEEK via UV-induced self-initiation and N-halamine grafting

**DOI:** 10.1016/j.jot.2026.101081

**Published:** 2026-03-19

**Authors:** Ziying Cheng, Yansheng Huang, Zhigang Zhao, Songnan Shao, Zitong Zhao, Xiaoshuai Peng, Baorong He, Zhen Chang

**Affiliations:** a Department of Spine Surgery, Xi'an Honghui Hospital, Xi'an Jiaotong University, Youyidong Road, Xi'an, Shaanxi, 710000, PR China; b Shanxi Medical University, Taiyuan, Shanxi, 030001, PR China; c Department of Ophthalmology, Xi'an Honghui Hospital, Xi'an Jiaotong University, Youyidong Road, Xi'an, Shaanxi, 710000, PR China; d The Eighth Affiliated Hospital, Sun Yat-sen University, Shenzhen, Guangdong, 518033, PR China

**Keywords:** Antibacterial property, Antibiotic-free, Biocompatibility, N-halamine, PEEK, Photo-induced self-initiation

## Abstract

**Background:**

Implant-associated infection remains a major challenge in orthopedic surgery. Although polyether ether ketone (PEEK) is widely used owing to its favorable mechanical properties and radiolucency, its intrinsic bioinertness renders it highly susceptible to bacterial colonization. Developing a durable, antibiotic-free antibacterial surface modification for PEEK with translational relevance remains clinically desirable.

**Methods:**

In this study, a covalently grafted N-halamine–functionalized PEEK surface (PEEK-*g*-PAM-NCl) was fabricated via a UV-induced self-initiated polymerization strategy. The antibacterial performance was systematically evaluated against *Staphylococcus aureus* and *Escherichia coli* using time–kill assays, live/dead staining, and antibacterial tests under protein-rich conditions. Biocompatibility and osteogenic potential were assessed in vitro using hBM-MSCs. In vivo antibacterial efficacy and biocompatibility were further investigated using subcutaneous implant-associated infection model in the C57BL/6J mice and femoral osteomyelitis model in the SD rat.

**Results:**

PEEK-*g*-PAM-NCl exhibited sustained and effective antibacterial activity against both Gram-positive and Gram-negative bacteria, achieving >98% bacterial reduction in vitro, including under protein-rich conditions. Time–kill assays revealed a pronounced time-dependent bactericidal effect. In vivo, PEEK-*g*-PAM-NCl significantly reduced bacterial burden, inflammatory infiltration, and infection-associated tissue damage compared with pristine PEEK, effectively preventing chronic osteomyelitis features. Importantly, no abnormal systemic inflammatory response or local tissue toxicity was observed. In vitro osteogenic assessments demonstrated that the antibacterial modification did not adversely affect osteogenic differentiation.

**Conclusion:**

This study demonstrates that covalent N-halamine functionalization endows PEEK with sustained, antibiotic-free antibacterial activity while preserving biocompatibility performance. PEEK-*g*-PAM-NCl represents a promising translational strategy for preventing implant-associated infections in orthopedic applications.

**The translational potential of this article:**

This study presents an antibiotic-free antibacterial surface modification strategy for PEEK implants based on UV-induced covalent grafting of N-halamine polymers. The robust antibacterial efficacy, preserved cytocompatibility, and supportive bone-related biological responses suggest potential applicability in preventing implant-associated infections in orthopedic settings. This work provides a feasible surface engineering approach that may be further optimized and translated toward clinically relevant load-bearing implants.

## Graphical abstract

A photo-induced self-initiation and chlorination approach enables the functionalization of PEEK with N-halamine groups (PEEK-*g*-PAM-NCl), imparting robust antibacterial activity while preserving biocompatibility. This antibiotic-free surface modification offers a promising strategy for reducing implant-associated infections in orthopedic applications.

**Figure:**
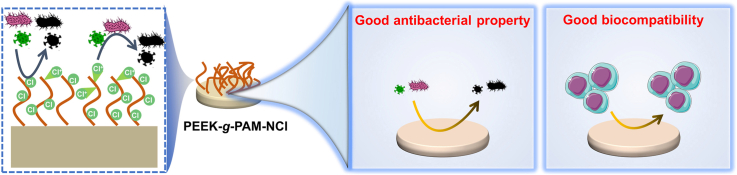

## 1 Introduction

**Fig. 1:**
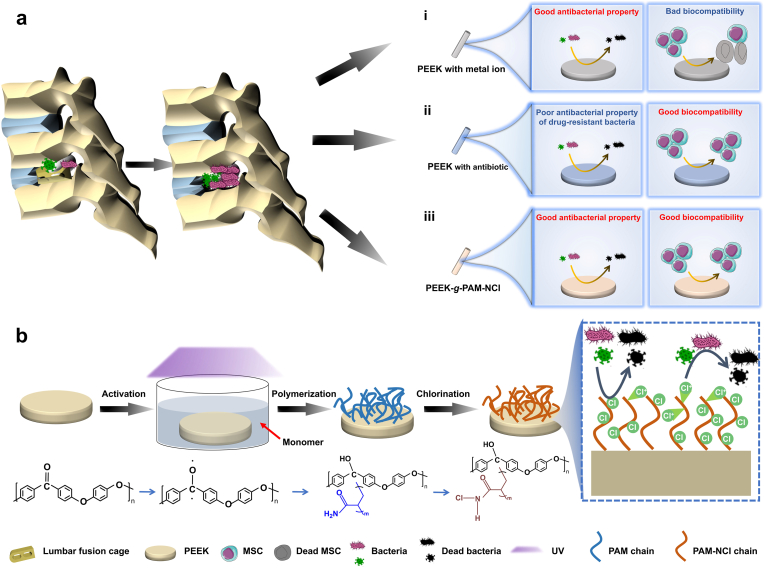
Overview of material properties and functional strategies. (a) Schematic illustration of infection associated with PEEK interbody fusion devices and corresponding modification strategies to address this issue, including (i) incorporation of antibacterial metal ions, (ii) incorporation of antibiotics, and (iii) our novel antibacterial functional coating strategy. Although the metal-ion modification strategy provides strong antibacterial activity, most incorporated ions are heavy metals with potential cytotoxicity. The antibiotic-based modification strategy exhibits good biocompatibility but remains ineffective against infections caused by drug-resistant bacteria. In contrast, our antibacterial functional coating strategy simultaneously ensures favorable biocompatibility and excellent antibacterial properties.(b) Schematic illustration of the preparation process of PEEK-g-PAM-NCl via UV-induced self-initiated grafting of polymer molecular brushes followed by chlorination, as well as the antibacterial performance of PEEK-g-PAM-NCl.

Polyetheretherketone (PEEK), as a representative aromatic semi-crystalline thermoplastic polymer, has been regarded as one of the next-generation “star” orthopedic implant materials, owing to its excellent mechanical properties that provide sufficient load-bearing capacity while mitigating stress shielding during implantation in bone tissues [[1], [2], [3]]. Nevertheless, with the increasing prevalence of degenerative and traumatic orthopedic disorders, the clinical demand for implants has grown steadily, accompanied by rising incidences of implant-associated complications. Among them, implant-associated infection (IAI) [4], characterized by progressive inflammation and bone destruction, represents one of the most severe challenges. The potential sources of infection include the surgical environment (e.g., operating room air, surfaces, surgical instruments, and medical staff attire), the patient's skin, and endogenous bacteria [5]. Current therapeutic strategies mainly rely on antibiotic administration, immune modulation, and, when necessary, surgical debridement and implant replacement (Fig. 1a), which together impose an enormous economic burden estimated at USD 3.3 billion annually [6]. Therefore, developing effective antibacterial modifications for orthopedic implants is an urgent and unmet clinical demand.

According to the “race for the surface” theory proposed by Gristina et al. [7], the outcome of implant integration largely depends on whether host cells or bacteria first colonize the implant surface. Once bacteria dominate, biofilm formation ensues, acting as a protective shield composed of extracellular polymeric substances such as proteins, polysaccharides, and nucleic acids. This biofilm not only protects bacteria from immune clearance and antimicrobial agents but also facilitates nutrient transfer and persistent colonization [8]. Antibacterial surface functionalization of PEEK has thus emerged as a promising strategy to inhibit biofilm formation and reduce infection. For instance, nanosilver or other metal-based coatings can impart strong antibacterial activity [9]; however, the potential cytotoxicity of released heavy metal ions limits their long-term in vivo applicability (Fig. 1a (i)). Similarly, antibiotic-loaded PEEK, such as minocycline-modified PEEK [10], often fails against drug-resistant bacterial strains, diminishing its clinical utility (Fig. 1a (ii)).

Herein, we report a novel modification strategy to endow PEEK with antibiotic-free antibacterial activity and excellent biocompatibility. Specifically, a UV-induced self-initiated in situ grafting of polyacrylamide (PAM) molecular brushes followed by chlorination was employed to construct a covalently bonded antibacterial functional coating on PEEK (Fig. 1a (iii)). Benefitting from the biocompatibility of PAM and the dual antibacterial mechanism of N-halamine functional groups (contact-killing and release-killing), the modified PEEK (PEEK-*g*-PAM-NCl) exhibited favorable cytocompatibility both in vitro and in vivo, while maintaining a high antibacterial efficiency of up to 98.4% even after 6 weeks of implantation (Fig. 1b). This quasi-nondestructive functionalization strategy offers a promising material-based alternative to mitigate implant-associated infections and improve the long-term clinical performance of PEEK-based implants.

## 2 Materials and methods

### 2.1 Materials

PEEK samples were purchased from Guangzhou Muran Biochemical Technology Co., Ltd (Guangzhou, China). For implantation in the C57BL/6J mouse subcutaneous biocompatibility model and subcutaneous implant-associated infection model, PEEK discs with a diameter of 10 mm and a thickness of 1 mm were used. For implantation in the SD rats femoral bone defect model and femoral bone osteomyelitis model, cylindrical shape (2 mm in diameter, 10 mm in height) were used. For pull-off test, cuboid-shaped specimens with dimensions of 4 mm × 10 mm × 20 mm were used. Unless otherwise specified, all other experiments were conducted with PEEK discs of 15 mm in diameter and 2 mm in thickness. Acrylamide (AM) monomer was obtained from Macklin (Shanghai, China); potassium iodide from Beichen (Tianjin, China); starch indicator from Macklin (Shanghai, China); and sodium thiosulfate from Aladdin (Shanghai, China). All chemical reagents were used as received without further purification.

The cells used in this study were human bone marrow-derived mesenchymal stem cells (hBM-MSCs, LONGBIO, Shanghai, China; 1 × 10^6^). The bacterial strains included *Staphylococcus aureus* (*S. aureus*, ATCC 25923, Guangdong Microbial Culture Collection Center, China) and *Escherichia coli* (*E. coli*, ATCC 25922, Guangdong Microbial Culture Collection Center, China).

### 2.2 Methods

#### 2.2.1 Preparation of PEEK samples

**PEEK-*g*-PAM:** PEEK samples (15 mm in diameter, 2 mm in thickness) were sequentially ultrasonically cleaned with acetone, ethanol, and deionized water for 15 min each. The discs were then dried in an oven at 60 °C under atmospheric pressure for 2 h. Acrylamide monomer (4 g) was dissolved in 20 mL deionized water by ultrasonic treatment for 5 min to obtain a precursor solution. The dried PEEK discs were placed in transparent glass dishes (6 cm in diameter, 2 cm in height), and 8 mL of the precursor solution was uniformly added to each dish. The surfaces were irradiated with a 400 W UV curing lamp at 365 nm with an intensity of 10 mW/cm^2^ positioned 6 cm above the PEEK surface for 45 min to initiate polymerization. The resulting polyacrylamide-coated PEEK discs were immersed in a 1000 mL beaker filled with deionized water for 8 h to remove unreacted monomers, with water replaced every 2 h. The samples were dried in an oven at 60 °C for 1 h to obtain PEEK grafted with polyacrylamide brushes (PEEK-*g*-PAM).

**PEEK-*g*-PAM-NCl:** The PEEK-*g*-PAM samples were immersed in a sodium hypochlorite solution (4% available chlorine) for 120 min to achieve N-halogenation for antibacterial functionalization. After treatment, the discs were rinsed five times with deionized water and air-dried to obtain PEEK grafted with antibacterial functional groups (PEEK-*g*-PAM-NCl).

#### 2.2.2 Structure characterization

Surface and cross-sectional microstructures were investigated with field-emission scanning electron microscope (S-4800, Hitachi, Japan, and Sigma 300, Zeiss, Germany). Elemental mapping was investigated with an Energy Dispersive X-ray Spectroscopy (EDS/EDX) (Sigma 300, Zeiss, Germany). Water contact angle was measured by a drop shape analyzer (DSA 100, Kruss, Germany; 3 μL per time, n = 3). The bonding strength (Fig. S1) was measured by pull-off test. Considering the thickness and grafted nature of the polymer layer, a shear-based adhesion evaluation [11] was employed to avoid bulk-coating failure dominating the measured adhesion strength.

#### 3.2.3 Chlorine content determination and stability

**Stability tests.** PEEK-*g*-PAM-NCl samples were placed on a conventional chemical laboratory bench at room temperature and kept static for predetermined periods, after which the chlorine content was measured. For ultraviolet (UV) stability evaluation, a UV curing lamp with a power of 35 W was positioned 6 cm above the PEEK-*g*-PAM-NCl samples to provide continuous UV irradiation at room temperature; the chlorine content was subsequently determined after specified exposure times. In addition, PEEK-*g*-PAM-NCl samples were immersed in phosphate-buffered saline (PBS) solution and incubated at 37 °C for designated durations, followed by measurements of chlorine content [12] and antibacterial activity.

According to the standard iodometry/thiosulfate titration procedure [13], we determined the Cl^+^ content through the following formula:

$$\text{Cl}+\left(p.p.m.\right)=\frac{C\times V\times 35.45}{W\times 2}\times 10^{6}$$

Among them, C and V represent the concentration (mol L-1) and volume (L) of thiosulfate consumed during the titration process, and W represents the weight (g) of the tested sample.

#### 2.2.4 Cell culture

**Cell culture.** hBM-MSCs were cultivated in a mixed medium of 10% fetal bovine serum (FBS, Tianhang, Zhejiang, China) along with 90% Dulbecco's modified Eagle's medium (DMEM, Gibco, USA), maintained at 37 °C and 5% CO_2_ atmosphere. Medium was refreshed every three days.

#### 2.2.5 In vitro biocompatibility

**Cell adhesion and morphology.** Disinfected PEEK samples were introduced into 24-well plates alongside hBM-MSCs (1 × 10^4^ cell mL^−1^) for 1 day. The cell-adhered samples were then fixed by 4% paraformaldehyde at 4 °C for 30 min, permeabilized with 0.1% Triton X-100 for 15 min and stained with Phalloidin (Beyotime, Shanghai, China) for 30 min and 4 ′, 6-diamino-2-phenylindole (DAPI, Beyotime, Shanghai, China) for 5 min for determining F-actin and cell nuclei, respectively. Finally, PEEK samples were observed by CLSM (LSM880, Zeiss, Germany).

**Cytotoxicity and vitality.** Cell Counting kit-8 (CCK8) was to detect the OD values of cells co-cultured with PEEK, PEEK-NCl and PEEK-pNCl on the 1st, 3rd and 7th days. Specifically, hBM-MSCs were seeded on the disinfected PEEK samples at a cell density of 5 × 10^4^ cell mL^−1^. Then, PEEK samples were co-cultivated with detection reagents (DMEM_10%FBS_: CCK8 = 10:1) for 1 h, and OD values at 450 nm were recorded on the aforementioned days.

**In vitro antioxidant performance.** The in vitro antioxidant activity of the PEEK samples was evaluated using the 2,2-diphenyl-1-picrylhydrazyl (DPPH·) assay. In brief, 1 mL of 0.1 mM (approximately 0.04 mg mL^−1^) DPPH· methanol solution was added to the surface of each sterilized sample, followed by incubation at 37 °C for 30 min. The supernatant was then collected and transferred to a 96-well plate, and the absorbance at 517 nm was measured. The DPPH· scavenging rate was calculated using the following formula:

$$\begin{array}{c} D\text{PPH}\cdot \;\text{scavenging}\left(\%\right)=\frac{\text{AB}‐\text{AS}}{\text{AB}}\times 100 \end{array}$$

Among them AB represents the absorbance of the DPPH· solution, and AS represents the absorbance of the DPPH· solution after reaction with the sample; methanol was used as the blank baseline, and the absorbance of the DPPH· methanol solution served as the control group.

#### 2.2.6 In vitro osteogenic assessments

**Expression of in vitro osteogenesis-related genes.** Real-time quantitative reverse transcription polymerase chain reaction (RT-qPCR) was conducted to assess osteogenesis-related gene expression on day 7 of osteogenic induction. Total RNA was extracted from hBM-MSCs cultured on different samples using an RNA rapid purification kit (ES Science, Shanghai, China) and reverse-transcribed into cDNA using an Evo M-MLV reverse transcription premix (AG, Hunan, China). RT-qPCR was performed using a SYBR Green Pro Taq HS premixed kit (including ROX; AG, Hunan, China) on an S7500 real-time PCR system (Thermo Fisher Scientific, USA). The expression levels of osteocalcin (OCN), osteopontin (OPN), and Runx-2 were calculated using the 2^−ΔΔCt^ method, with glyceraldehyde-3-phosphate dehydrogenase (GAPDH) serving as the housekeeping gene. Primer sequences are listed below.

**Table:** 

Genes	Primers
OCN [14]	Froward: 5′-GAGGGCAGTAAGGTGGTGAA-3′
	Reverse: 5′-GTCCGCTAGCTCGTCACAAT-3′
OPN [15]	Froward: 5′-AAGTTTCGCAGACCTGACATC-3′
	Reverse: 5′-GGCTGTCCCAATCAGAAGG-3′
Runx-2[14]	Froward: 5′-GCCGGGAATGATGAGAACTA-3′
	Reverse: 5′-GGACCGTCCACTGTCACTTT-3′
GAPDH [14]	Froward: 5′-TGACCTCAACTACATGGTCTACA-3′
	Reverse: 5′-CTTCCCATTCTCGGCCTTGTACA-3′

**ALP staining and ALP activity quantification.** PEEK samples were placed in 24-well plates and co-cultured with human bone marrow–derived mesenchymal stem cells (hBM-MSCs) at a density of 5 × 10^4^ cells per well to induce osteogenic differentiation. On day 7 after osteogenic induction, the samples were fixed with 4% paraformaldehyde at 4 °C for 15 min and subsequently stained with BCIP/NBT (Beyotime, Shanghai, China) for 20 min in the dark. For ALP activity quantification, proteins were extracted using radioimmunoprecipitation assay (RIPA) buffer supplemented with a protease inhibitor (PMSF), and ALP activity was determined using a colorimetric assay kit (Elabscience, Wuhan, China) according to the manufacturer's instructions.

**Alizarin Red S (ARS) staining and semi-quantitative analysis.** PEEK samples were seeded in 24-well plates and co-cultured with hBM-MSCs (5 × 10^4^ cells per well) under osteogenic induction conditions. On day 14, the samples were fixed with 4% paraformaldehyde at 4 °C for 30 min and stained with Alizarin Red S solution (Cyagen, Suzhou, China) for 30 min to visualize mineralized calcium nodules. For semi-quantitative analysis, 1 mL of 10% (w/v) cetylpyridinium chloride (CPC; Sigma–Aldrich, USA) in sodium phosphate buffer (pH 7.0) was added to each well and gently shaken for 60 min to dissolve the bound dye. The optical density (OD) was measured at 562 nm.

#### 2.2.7 In vitro antibacterial assessments

**Release Antibacterial and Contact Antibacterial Tests.** The antibacterial ability was firstly evaluated by counting colony-forming units (CFU) on broth agar plates with *S. aureus* (ATCC25923, Guangdong Provincial Microbial Collection Center) and *E. coli* (ATCC25922, Guangdong Provincial Microbial Collection Center). The specific steps were as followed: Disinfected samples were inserted into 24-well plate, and co-cultured with 500 μL bacterial fluid each well as a density of 1 × 10^5^ cell mL^−1^ at 37 °C for 12 h. To detect the release antibacterial rate, the supernatants were diluted 10,000-fold, and seeded on broth agar plates. To detect the contact antibacterial rate, bacteria adhered on the samples were collected by immersing in 1 mL PBS each well. The supernatants were diluted 10,000-fold after ultrasonic treatment, and seeded on broth agar plates. After 12 h cultivation, the CFU was counted in the Image J software, and the release and contact antibacterial rates were calculated through the following formula:

$$\text{Antibacterial}\;\text{rate}\;\left(\%\right)=\frac{\text{CFU}\;\text{of}\;\text{control}\;\text{group}‐\text{CFU}\;\text{of}\;\text{experimental}\;\text{group}}{\text{CFU}\;\text{of}\;\text{control}\;\text{group}}\times 100$$

Among the samples, PEEK belongs to the control group, while PEEK-*g*-PAM-NCl belongs to the experimental group.

Notably, the same procedures were additionally carried out in LB medium containing 10% fetal bovine serum (FBS) to simulate a protein-rich environment, with bacterial plating and quantitative analysis performed at 12 and 24 h, respectively.

**Bacterial live/dead staining and quantification.** Fluorescent labeling was used to analyze the total antibacterial ability. Disinfected samples were inserted into 24-well plate, and co-cultured with 500 μL bacterial fluid each well (*S. aureus* and *E. coli*) at a density of 1 × 10^5^ cell mL^−1^ at 37 °C for 24 h. The samples were taken out under aseptic operation, the supernatants were laid aside for 2 h, stained for 15 min in the dark with LIVE/DEAD™ BacLight™ Bacterial Viability Kit (Thermo Fisher, 1 kit, USA), and observed under a fluorescence microscope (LSM 780, Zeiss, Germany). Specifically, at least five random microscopic fields were analyzed per sample, with a minimum of three independent samples per group (n = 3). The acquired fluorescence images were analyzed using ImageJ software. Based on the grayscale values obtained from the fluorescence images, the proportion of the red fluorescence-labeled area was calculated according to the following formula.

$$\begin{array}{c} \text{Red}\;\text{fluorescence}-\text{labeled}\;\text{area}\;\text{proportion}\left(\%\right)=\frac{\text{The}\;\text{area}\;\text{of}\;\text{red}\;\text{fluorescence}}{\text{The}\;\text{total}\;\text{of}\;\text{red}\;\text{and}\;\text{greeen}\;\text{fluorescence}}\times 100 \end{array}$$

**Bacterial adhesion.** 1 × 10^5^ CFU mL^−1^ (500 μL each well) of *S. aureus* or *E. coli* were co-cultured with samples at 37 °C for 24 h. Then, PEEK samples were cleaned by PBS, fixed with 4 % paraformaldehyde at 4 °C for 30 min, dehydrated with gradient ethanol and freeze-dried in sequence. Finally, the samples were observed under field emission scanning electron microscope (S-4800, Hitachi, Japan).

**Bacterial biofilm formation.** 1 × 10^6^ CFU mL^−1^ (500 μL each well) of *S. aureus* or *E. coli* were co-cultured with samples at 37 °C for 24 h. Subsequently, PEEK samples were extracted under sterile operation, cleaned by PBS, and stained with 1 mL 0.5% crystal violet staining solution each well (Beyotime, Shanghai, China) for 15 min. Finally, the biofilm was dissolved with 1 mL methanol each well, and the OD value was measured at 590 nm.

**Inhibition zone assay.** The inhibition zone assay was used to evaluate the effect of antimicrobial group release or leachable antibacterial substances from the material surface on surrounding bacteria. In brief, 100 μL of adjusted bacterial suspension (concentration 1 × 10^5^ CFU mL^−1^) was added to the surface of a solid agar plate and spread evenly. The material was then placed in the center of the agar plate. The plate was incubated at 37 °C for 12 h. The diameter of the inhibition zone was measured using Image J software.

**Antimicrobial performance testing.** To further evaluate the broad-spectrum antibacterial and antifungal properties of each sample group, representative microorganisms were selected, including bacteria—*S. aureus* (*S. aureus*), *E. coli* (*E. coli*), *Pseudomonas aeruginosa* (*P. aeruginosa*); antibiotic-resistant bacteria—methicillin-resistant *S. aureus* (MRSA); and fungus—*Candida albicans* (*C. albicans*). Broad-spectrum antibacterial and antifungal assessments were conducted at the Guangzhou Institute of Microbiology. In brief, each sample group was co-cultured with different microorganisms in culture bottles at 37 °C for 18 h. After 18 h, bacterial/fungal counts in the culture bottles were measured and plated for colony counting.

**Time-kill assay.** Time–kill assays were performed to evaluate the antibacterial activity of PEEK-*g*-PAM-NCl against *S. aureus* and *E. coli*. Briefly, bacterial suspensions were prepared with an initial concentration of 1 × 10^5^ CFU mL^−1^. Sterilized PEEK and PEEK-*g*-PAM-NCl samples were incubated with the bacterial suspensions at 37 °C under shaking conditions. At predetermined time points (0, 2, 4, 8, 12, and 24 h), aliquots were collected from each group and serially diluted at different dilution factors (10, 100, 100, 1000, 10,000, and 10,000-fold, respectively). Subsequently, 20 μL of each diluted suspension was spread onto agar plates and incubated at 37 °C for 24 h. Colony-forming units (CFUs) were counted, and bacterial viability was expressed as log_10_ (CFU mL^−1^). All experiments were performed in triplicate.

#### 2.2.8 In vivo biocompatibility assessment

**Construction of Subcutaneous Tissue Compatibility Model.** A subcutaneous implantation model was established using C57BL/6J mice to evaluate the tissue compatibility of the materials. Male C57BL/6J mice (8 weeks old) were randomly divided into two groups (n = 6 per group). Following anesthesia with an intraperitoneal injection of sodium pentobarbital (50 mg kg^−1^), the surgical area was shaved and disinfected. A 1 cm longitudinal incision was made, and the skin and subcutaneous layers were bluntly dissected to expose the plane above the fascia. Implants (10 mm in diameter, 1 mm in thickness) of either PEEK or PEEK-*g*-PAM-NCl were implanted subcutaneously, followed by layered closure.

One weeks after surgery, all mouse were sacrificed with an overdose of pentobarbital (Sigma–Aldrich, USA). The peri-implant soft tissues (epidermal and subcutaneous layers) and major organs (heart, liver and kidney) were harvested and fixed in 4% paraformaldehyde for 24 h. Hematoxylin and eosin (H&E) staining and CD68 immunohistochemical (1:200) staining were performed to evaluate the inflammatory infiltration of the tissues. The peri-implant soft tissues and major organs section was performed by Hubei BIOSSCI Biotechnology Co., Ltd.

**Construction of the femoral bone defect model.** Ten-week-old male SD rats were randomly assigned to three groups (n = 9 per group; total n = 18), and a left femoral bone defect model was established in all animals. Briefly, following intraperitoneal anesthesia with pentobarbital, the surgical site was disinfected with povidone-iodine, and a medial parapatellar incision was made. The patella was laterally dislocated to expose the femoral condyles, after which the medullary canal was sequentially reamed using 22 G, 20 G, and 18 G needles, followed by a Kirschner wire (diameter = 2.2 mm). The prepared samples were then inserted into the medullary cavity, and the wound was closed in layers.

At 3 days postoperatively, blood samples were collected from three rats in each group for routine hematological analysis. At 6 weeks post-implantation, the remaining animals were euthanized by an overdose of pentobarbital (Sigma–Aldrich, USA). For radiological evaluation, six rats (three per group) were randomly selected, fixed in 10% neutral buffered formalin, and scanned using a high-resolution micro-computed tomography (micro-CT) system (Inveon, Siemens, Germany) at a voltage of 50 kV, a current of 500 μA, and a voxel size of 10 μm. A cylindrical region of interest (ROI; diameter 2.2 mm, height 10 mm) adjacent to the femoral condyle was defined. Sagital, coronal and axial sections, as well as three-dimensional reconstructed images, were generated using RadiAnt DICOM Viewer software. Quantitative parameters, including bone volume/total volume (BV/TV), bone surface area/bone volume (BS/BV), trabecular number (Tb.N), and trabecular thickness (Tb.Th), were calculated.

H&E staining was performed to assess inflammatory cell infiltration, while Masson trichrome staining was used to evaluate new bone formation around the implants. Histological sectioning of femoral tissues was conducted by Hubei BIOSSCI Biotechnology Co., Ltd.

#### 2.2.9 In vivo antibacterial assessment

**Construction of Subcutaneous Tissue implant-associate Infection Model.** C57BL/6J mice were used to assess the in vivo antibacterial activity. Male C57BL/6J mice (8 weeks old) were randomly divided into two groups (n = 6 per group). Following anesthesia via intraperitoneal injection of sodium pentobarbital (50 mg kg^−1^), the surgical site was shaved and disinfected. A 1 cm longitudinal incision was made, and the epidermal and subcutaneous layers were carefully separated to expose the fascia. A 10 μL suspension of *S. aureus* (1 × 10^5^ CFU mL^−1^) was introduced into the exposed dorsal pocket using a micropipette and allowed to equilibrate for 1 min. Subsequently, implants (10 mm in diameter, 1 mm in thickness) of PEEK or PEEK-*g*-PAM-NCl were implanted into the pocket, followed by layered closure of the incision.

One weeks after surgery, all mouse were sacrificed with an overdose of pentobarbital (Sigma–Aldrich, USA). And the retrieved implants were immediately immersed in 1 mL sterile PBS in 15 mL centrifuge tubes and subjected to ultrasonication for 3 min. Subsequently, 20 μL of the PBS suspension was plated onto agar plates for bacterial colony counting. The plates were incubated in a bacterial incubator for 12 h, after which colony images were captured and the antibacterial rate was calculated. Meanwhile, the surrounding soft tissues adjacent to the implants from each group were fixed in 4% paraformaldehyde for 24 h for histological analysis. Hematoxylin and eosin (H&E) staining was performed to evaluate the inflammatory infiltration of the tissues, and Giemsa staining was performed to evaluate the number of bacteria in the tissues. The peri-implant soft tissues section was performed by Hubei BIOSSCI Biotechnology Co., Ltd.

**Construction of the femoral osteomyelitis model.** Ten-week-old male SD rats were randomly allocated into three groups (n = 6 per group; total n = 18), and a left femoral osteomyelitis model was established in all animals. Briefly, after intraperitoneal anesthesia with pentobarbital, the surgical field was prepared and disinfected with povidone–iodine, followed by a medial parapatellar incision. The patella was laterally dislocated to expose the medial and lateral femoral condyles. The medullary canal was sequentially enlarged using 22 G, 20 G, and 18 G needles, followed by a Kirschner wire (diameter = 2.2 mm). Subsequently, a *S. aureus* suspension (10 μL, 1 × 10^5^ CFU per rat) was injected into the medullary cavity. The corresponding implants were then inserted, and the wound was closed in layers.

At 6 weeks postoperatively, all rats were euthanized by an overdose of pentobarbital (Sigma–Aldrich, USA). For radiological evaluation, the steps were the same as section 2.2.8.

For bacterial analysis, another six rats (three per group) were randomly selected, and the implants were aseptically retrieved and immersed in 10 mL of PBS followed by ultrasonic treatment. Subsequently, 20 μL of the resulting suspension was plated onto broth agar plates for colony counting, and the antibacterial rate was calculated.

For histological evaluation, femoral specimens were decalcified in 10% EDTA for 4 weeks. H&E staining was performed to assess inflammatory cell infiltration. While Giemsa staining was used to evaluate bacterial presence and quantitative analysis of inflammatory cell density and inflammatory area proportion was conducted using ImageJ software. Masson trichrome staining was further performed to assess new bone formation surrounding the implants.

Finally, a basic medical science expert and a clinical pathology expert independently performed histopathological inflammatory scoring based on the radiological and histological findings. The HOES score [16] was evaluated according to four parameters: inflammatory cell infiltration, medullary cavity structural destruction, fibrosis/granulation tissue formation, and necrotic areas. Each parameter was scored on a 0–3 scale, with higher scores indicating greater severity.

### 2.3 Data collection and statistical analysis

All data are presented as mean ± standard deviation (SD). An independent-samples t-test was used to evaluate statistical differences between two groups, while one-way ANOVA followed by Bonferroni post hoc test was applied for multiple comparisons among three or more groups. Statistical significance was defined as *p* < 0.05 (*p* < 0.05, ∗*p* < 0.01, ∗∗*p* < 0.001, ns: not significant). All statistical analyses were performed using SPSS version 25.0 (IBM Corp., Armonk, NY, USA).

## 3 Results

### 3.1 Preparation and structural characterization

**Fig. 2:**
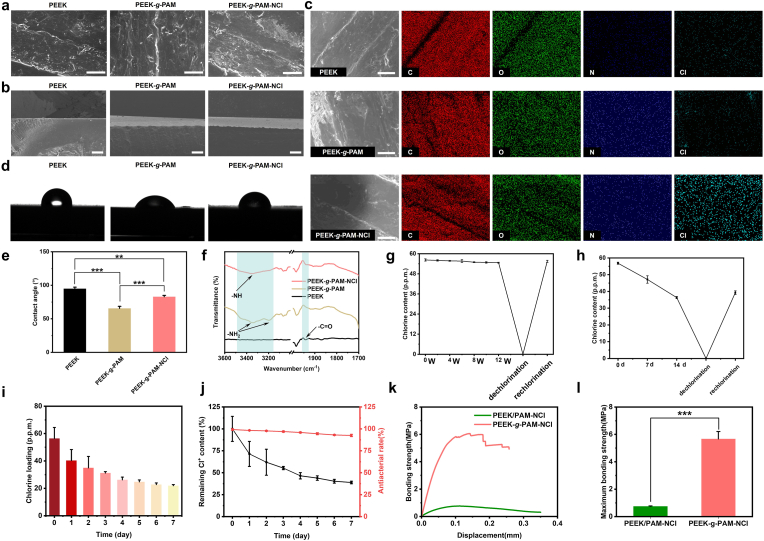
**Structural characterizations**. SEM images of the surface (a) and cross-sectional (b) microstructure of PEEK, PEEK-*g*-PAM, and PEEK-*g*-PAM-NCl (scale bars = 25 μm for figure a, and scale bars = 120 μm for figure b). (c) SEM image of PEEK, PEEK-*g*-PAM and PEEK-*g*-PAM-NCl with the corresponding representative elemental mapping (C, O, N and Cl, scale bars = 10 μm). The photos (d) and analysis (e) of water contact angles (n = 3) of PEEK, PEEK-*g*-PAM, and PEEK-*g*-PAM-NCl. (f) The ATR-FTIR spectra of PEEK, PEEK-*g*-PAM, and PEEK-*g*-PAM-NCl. Time-dependent chlorine content and re-chlorination curves of PEEK-*g*-PAM-NCl under (g) ambient laboratory conditions and (h) UV irradiation (n = 3). (i) Cl^+^ content of PEEK-*g*-PAM-NCl immersed in PBS for different times (n = 3) (j) Remaining Cl^+^ content of PEEK-*g*-PAM-NCl in PBS and corresponding contact antibacterial rate against *S. aureus* at different times (n = 3) (k) Bonding strength-displacement curves of PEEK/PAM-NCl and PEEK-*g*-PAM-NCl in a partially dried hydrogel state and (l) Maximum bonding strength of PEEK/PAM-NCl and PEEK-*g*-PAM-NCl (n = 3). The data are mean ± SD, ∗*p* < 0.05, ∗ ∗*p* < 0.01, ∗ ∗ ∗*p* < 0.001, ns means not statistically significant.

Based on the photo-induced self-initiation strategy of PEEK [17], the benzophenone-like units within PEEK undergo bond cleavage under UV irradiation, generating semipinacol radicals. These radicals initiate the cleavage of C=C double bonds in acrylamide (AM) monomers, thereby triggering polymerization and simultaneously grafting the resulting polyacrylamide (PAM) chains onto the PEEK surface. As demonstrated in the surface and cross-sectional morphologies (Fig. 2a and b), the obtained modified PEEK (PEEK-*g*-PAM) exhibits a continuous PAM layer with a thickness of approximately 100 μm.

Furthermore, abundant primary amine groups on the PAM molecular brush layer were reacted with sodium hypochlorite, converting N–H bonds on the side chains into N–Cl bonds via amide coupling. As shown in Fig. 2a and b, the resultant antibacterial PEEK (PEEK-*g*-PAM-NCl) successfully maintains the continuous polymer layer with a consistent thickness of about 100 μm.

To further clarify the distribution of functional surface elements, elemental mapping analyses were performed (Fig. 2c). For pristine PEEK, the characteristic elements (C and O) display strong signals consistent with its inherent surface morphology. In comparison, PEEK-*g*-PAM exhibits markedly enhanced N signals, confirming the successful grafting of high-molecular-weight PAM. After chlorination, PEEK-*g*-PAM-NCl demonstrates a significant increase in Cl element signals, indicating the abundant introduction of N–Cl groups across the coating.

Surface wettability was further evaluated by water contact angle measurements (Fig. 2d and e). Owing to ether, ketone, and aromatic structures, pristine PEEK shows a hydrophobic angle of 94.8°. After PAM grafting, the introduction of amine groups reduces the contact angle to 65.6°, conferring hydrophilicity. Subsequent chlorination to form N–Cl bonds slightly increase the contact angle to 82.8°, while still maintaining an enhanced hydrophilic property compared with pristine PEEK.

The ATR-FTIR spectra was conducted to further verify the grafting of functional groups (Fig. 2f). Pristine PEEK exhibits a weak absorption band around 1936 cm^−1^, which has been reported [17] in some aromatic polymers and is generally attributed to overtone or combination vibrations rather than a typical carbonyl stretching mode. After UV-induced grafting of PAM, the overall FTIR spectral features of PEEK remain largely preserved, while new absorption bands associated with amide groups become evident, indicating successful surface grafting without disruption of the PEEK backbone. Specifically, in PEEK-*g*-PAM, characteristic amide-related bands appear, including N–H stretching vibrations at 3351 cm^−1^ and 3197 cm^−1^, consistent with the presence of primary amine functionalities [18]. Following chlorination, PEEK-*g*-PAM-NCl shows the disappearance of the 3197 cm^−1^ band while retaining the 3351 cm^−1^ band, suggesting partial substitution of N–H bonds by N–Cl bonds [19]. These spectral changes collectively confirm the successful functionalization of the PEEK surface with N-halamine groups.

The stability of grafted functional groups was monitored under different environments (Fig. 2g and h). Under ambient laboratory conditions for 12 weeks, the surface chlorine content decreased from 56.26 ppm to 54.65 ppm, retaining 97.1% of its initial value with only 2.9% loss. In contrast, under UV exposure for 2 weeks, chlorine content dropped sharply from 56.9 ppm to 36.3 ppm, retaining only 63.9% of its initial value with a 36.1% loss.

Considering the reversible transformation between N–Cl and N–H bonds, rechlorination experiments were performed on samples after environmental exposure (Fig. 2g and h). Under ambient conditions, chlorine content was restored to 55.5 ppm, retaining 98.6% of the initial level. However, after UV exposure, rechlorination restored chlorine content to only 39.3 ppm, corresponding to 69.1% of the original level.

To evaluate the stability and sustained antibacterial performance of PEEK-*g*-PAM-NCl under physiological conditions, as shown in Fig. 2i and j, the chlorine content in a simulated physiological PBS environment gradually decreased from 56.40 p.p.m. to 21.89 p.p.m. over 7 days, indicating a controlled and sustained release behavior with a cumulative loss of approximately 61.19%. Notably, despite this decrease, PEEK-*g*-PAM-NCl still maintained a high antibacterial efficiency of 92.5%.

The inherent chemical inertness of the PEEK surface often results in poor bonding strength. To evaluate the bonding strength of PEEK-*g*-PAM-NCl, non-covalently grafted PEEK/PAM-NCl was used as a control, and the adhesion strength was measured using the pull-off method. As shown in Fig. 2k and l, the covalently bonded PEEK-*g*-PAM-NCl exhibited a significantly higher bonding strength (∼5.67 MPa) compared with the non-covalently bonded PEEK/PAM-NCl (∼0.75 MPa).

### 3.2 In vitro biocompatibility and pro-osteogenic effects

**Fig. 3:**
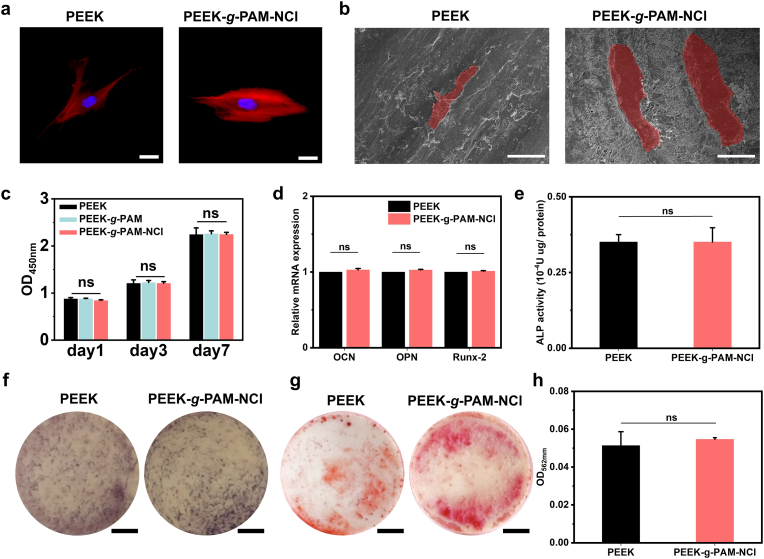
**In vitro biocompatibility and pro-osteogenic effects**. (a) Morphologies of hBM-MSCs co-cultured on the surface of PEEK and PEEK-*g*-PAM-NCl after 1 day (red for F-actin, blue for nuclei; scale bars = 25 μm; n = 3). (b) Cell morphology of hBM-MSCs co-cultured with PEEK and PEEK-*g*-PAM-NCl for 24 h under SEM (hBM-MSCs rendered in red; scale bars = 50 μm). (c) CCK-8 assay results of hBM-MSCs co-cultured with PEEK, PEEK-*g*-PAM, and PEEK-*g*-PAM-NCl for 1, 3, and 7 days (n = 3). (d) Relative mRNA expression of typical osteogenesis-related genes after hBM-MSCs co-cultured on the surface of PEEK and PEEK-*g*-PAM-NCl for 7 days in osteogenic medium (n = 3). (e) Quantitative analysis of ALP activity of hBM-MSCs co-cultured on the surface of PEEK and PEEK-*g*-PAM-NCl after 7 days in osteogenic medium (n = 3). (f) Digital photos of ALP staining (scale bars = 2 mm) of hBM-MSCs co-cultured on the surface of PEEK and PEEK-*g*-PAM-NCl after 7 days in osteogenic medium (n = 3). (g) Digital photos of Alizarin Red S staining (scale bars = 2 mm; n = 3) and (h) semi-quantitative analysis of hBM-MSCs co-cultured on the surface of PEEK, and PEEK-*g*-PAM-NCl after 14 days in osteogenic medium (n = 3). The data are mean ± SD, ∗*p* < 0.05, ∗ ∗*p* < 0.01, ∗ ∗ ∗*p* < 0.001, ns means not statistically significant.

The influence of the surface modification strategy on cellular responses was assessed by observing hBM-MSC morphology on PEEK and PEEK-*g*-PAM-NCl. As shown in Fig. 3a, hBM-MSCs cultured on PEEK exhibited a normal spindle-shaped morphology, with well-organized cytoskeletal filaments and intact elliptical nuclei with clearly defined edges. No abnormal structural features were observed. Similarly, hBM-MSCs on PEEK-*g*-PAM-NCl displayed comparable cytoskeletal and nuclear morphology to the PEEK group. Notably, the average cell length increased from 102 μm on PEEK to approximately 130 μm on PEEK-*g*-PAM-NCl.

To further examine the interactions between cells and the modified surface, hBM-MSCs were co-cultured with PEEK and PEEK-*g*-PAM-NCl, and cytoskeletal staining was applied to improve visualization against the background. As shown in Fig. 3b, cells on both surfaces maintained typical spindle-shaped contours; however, hBM-MSCs on PEEK-*g*-PAM-NCl exhibited greater spreading, with filopodia-like extensions protruding from the cell membrane. Moreover, a higher cell density was observed on the PEEK-*g*-PAM-NCl surface under the same magnification field.

Cell viability and cytotoxicity were further evaluated using the CCK-8 assay, which relies on the metabolic conversion of WST-8 into an orange formazan product by mitochondrial dehydrogenases [20]. As shown in Fig. 3c,S2, the optical density (OD) values of PEEK-*g*-PAM were slightly higher than those of PEEK, while PEEK-*g*-PAM-NCl showed a minor decrease compared with PEEK-*g*-PAM. Importantly, no statistically significant differences were detected among PEEK, PEEK-g-PAM, and PEEK-*g*-PAM-NCl at any time point, indicating that the surface modifications did not adversely affect hBM-MSC metabolic activity or viability.

Implantation of orthopedic devices inevitably involves invasive surgical procedures that may induce local oxidative stress and transient generation of reactive oxygen species (ROS) [21]. Under pathological conditions, accumulated ROS are always associated with oxidative stress, subsequently causing various damages to DNA, lipids, and proteins, leading to reduced cell proliferation, enhanced apoptosis, increased bacterial growth, and impaired wound healing [22].However, the present N-halamine–functionalized system was designed primarily for antibacterial purposes rather than for antioxidant protection.

To clarify whether the surface modification imparted any intrinsic radical-scavenging ability, the antioxidant capacity of the materials was evaluated using the DPPH· assay. As shown in Fig. S3, PEEK-*g*-PAM exhibited a minimal free radical scavenging rate of 1.21%, while PEEK-*g*-PAM-NCl showed a near-zero (−1.54%) value. No statistically significant differences were observed among the three groups.

The negligible or near-zero DPPH scavenging values indicate that neither PEEK nor its modified counterparts possess meaningful antioxidant activity, which is consistent with the oxidative nature of N-halamine chemistry. Therefore, the antibacterial performance of PEEK-*g*-PAM-NCl is unlikely to be associated with ROS scavenging, but rather attributed to oxidative chlorine species and contact-mediated bactericidal mechanisms.

In addition, the osteogenic-related effects of PEEK-*g*-PAM-NCl were evaluated. As shown in Fig. 3d, after 7 days of induction in osteogenic medium, quantitative PCR (qPCR) analysis of osteogenic markers revealed that the expression levels of OCN, OPN, and Runx-2 in PEEK-*g*-PAM-NCl were slightly increased compared with those in PEEK; however, no statistically significant differences were observed. Consistent with the qPCR results, further quantitative alkaline phosphatase (ALP) analysis (Fig. 3e), ALP staining (Fig. 3f), Alizarin Red S (ARS) staining (Fig. 3g), and the corresponding semi-quantitative analysis (Fig. 3h) showed similar trends.

### 3.3 In vitro antibacterial assessment

**Fig. 4:**
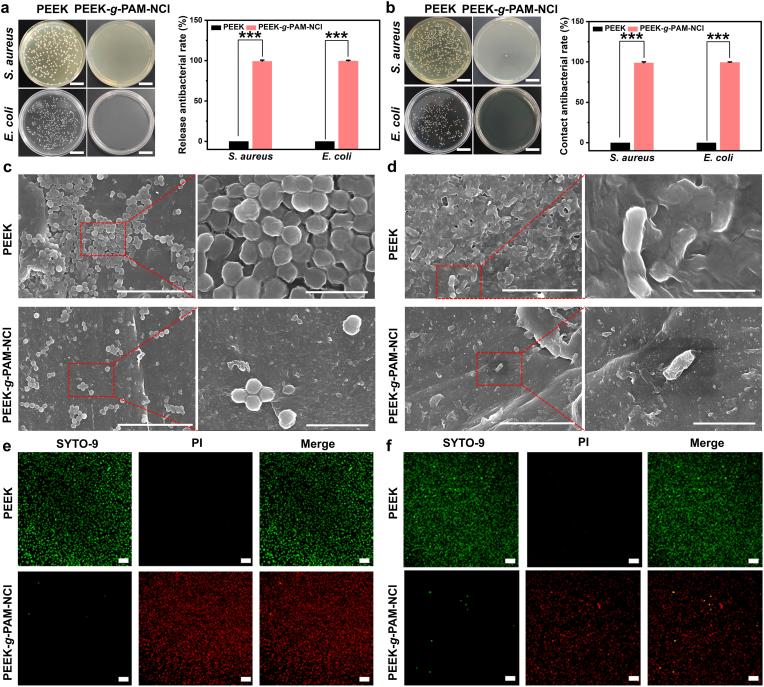
**In vitro antibacterial assessment**. (a) Images of bacterial colony-forming units by *S. aureus* and *E. coli* in the supernatants of PEEK and PEEK-*g*-PAM-NCl (scale bars = 2 cm), and release antibacterial rates against *S. aureus* and *E. coli* of PEEK and PEEK-*g*-PAM-NCl. (b) Images of bacterial colony-forming units by *S. aureus* and *E. coli* grown on PEEK and PEEK-*g*-PAM-NCl (scale bars = 2 cm) and contact antibacterial rates against *S. aureus* and *E. coli* of PEEK and PEEK-*g*-PAM-NCl. SEM images of bacterial morphologies of *S. aureus* (c) and *E. coli* (d) on PEEK and PEEK-*g*-PAM-NCl (left scale bars = 10 μm, right scale bars = 2 μm). The bacterial live/dead fluorescence staining showed the (e) *S. aureus* and (f) *E. coli* live/dead distribution on PEEK and PEEK-*g*-PAM-NCl (green represents live bacteria, red represents dead bacteria, yellow represents live bacteria with damaged cell membranes; scale bar = 100 μm). The data are mean ± SD, ∗*p* < 0.05, ∗ ∗*p* < 0.01, ∗ ∗ ∗*p* < 0.001, ns means not statistically significant.

Based on the gradual dissociation of N-halamine functional groups under moist physiological conditions [23], grafted N-halamine materials are capable of releasing antibacterial active species in situ to achieve potent bactericidal effects. After confirming the successful grafting of N-halamine groups on PEEK surfaces by standard iodometric titration, the release antibacterial activity was evaluated via dilution plating. *S. aureus* (*S. aureus*) was selected as the representative Gram-positive strain, and *E. coli* (*E. coli*) as the representative Gram-negative strain. As shown in Fig. 4a, abundant colonies of *S. aureus* and *E. coli* were observed on pristine PEEK, whereas almost no colonies were visible on PEEK-*g*-PAM-NCl. Quantitative analysis (Fig. 4a) further confirmed that PEEK-*g*-PAM-NCl exhibited striking release antibacterial activity, with 99.2% and 99.6% bactericidal rates against *S. aureus* and *E. coli*, respectively.

During the dissociation equilibrium process, a fraction of N-halamine groups remained on the material surface, thereby exerting sustained antibacterial effects against bacteria in direct contact. Following removal of the bacterial suspension, bacterial adhesion on sample surfaces was further examined. As shown in Fig. 4b, dense bacterial colonies were detected on PEEK, while PEEK-*g*-PAM-NCl showed a remarkable reduction in colony number. Quantitative statistics (Fig. 4b) demonstrated excellent contact antibacterial activity of PEEK-*g*-PAM-NCl, achieving 98.9% and 99.5% bactericidal rates against *S. aureus* and *E. coli*, respectively. In addition, the release antibacterial effects of PEEK and PEEK-*g*-PAM-NCl were evaluated using the in vitro inhibition zone assay. As shown in Fig. S4, under *S. aureus* conditions, no obvious sterile zone was observed around the PEEK group, whereas a clear sterile zone with a diameter of approximately 26.61 mm was observed around PEEK-*g*-PAM-NCl. Similarly, under *E. coli* conditions, compared with the PEEK group, a distinct sterile zone with a diameter of approximately 30.26 mm was observed around PEEK-g-PAM-NCl. The inhibition zones observed for PEEK-*g*-PAM-NCl (26–30 mm) are consistent with previously reported N-halamine–based antibacterial materials [24].

The anti-adhesion effect of the materials was further examined by SEM after 24 h bacterial co-culture. As shown in Fig. 4c and d, large numbers of intact *S. aureus* and *E. coli* adhered on PEEK surfaces, whereas bacterial attachment was substantially reduced on PEEK-*g*-PAM-NCl. Higher magnification SEM images revealed that bacteria on PEEK maintained smooth, intact morphologies, while residual bacteria on PEEK-*g*-PAM-NCl displayed deformation morphology suggestive of cell damage.

As PEEK-based orthopedic implants are intrinsically vulnerable to bacterial contamination during implantation [25], comprehensive antimicrobial evaluation against diverse representative microbes is essential. As shown in Fig. S5, the numbers of *E. coli*, *S. aureus*, *P. aeruginosa*, *C. albicans*, and MRSA were all significantly reduced on PEEK-*g*-PAM-NCl compared to pristine PEEK. Furthermore, enumeration of bacteria in co-culture media (Table S1) demonstrated that after 18 h incubation, the residual bacterial counts in the PEEK-*g*-PAM-NCl group were no colonies detected, confirming a broad-spectrum antimicrobial efficiency exceeding 99%.

**Fig. 5:**
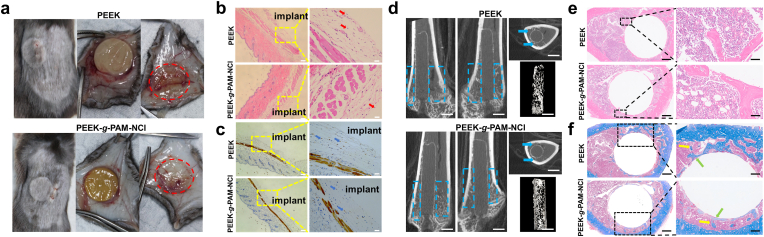
**In vivo biocompatibility properties**. After 1 week of implantation of PEEK and PEEK-*g*-PAM-NCl in C57BL/6J mice, (a) digital photographs of the dorsal skin, tissues surrounding the implants, and dorsal skin after removal of the implants; (b) H&E staining results; and (c) CD68 immunohistochemical staining results (red arrows indicate neutrophils, blue arrows indicate macrophages; left scale bars = 100 μm, right scale bars = 20 μm). After 6 weeks of implantation of PEEK and PEEK-*g*-PAM-NCl in SD rats, (d) Micro-CT scanning of femurs implanted with PEEK and PEEK-*g*-PAM-NCl and the corresponding reconstructed 3D images in the rat femoral bone defect model (the blue dashed box and blue arrow point to the bone trabeculae around the implant; scale bars = 2 mm). (e) H&E staining (left scale bars = 500 μm, right scale bars = 50 μm) and (f) Masson trisome staining (yellow arrow points to the trabecular bone, and the green arrow points to the newly formed bone around the implant; left scale bars = 500 μm, right scale bars = 250 μm) of peri-implanted femur in PEEK and PEEK-*g*-PAM-NCl groups in the rat femoral bone defect model.

To directly visualize bacterial viability, fluorescence staining with SYTO-9 and PI was employed, where SYTO-9 permeates all bacterial membranes and labels nucleic acids green, while PI penetrates only compromised membranes, producing red fluorescence [26]. As shown in Fig. 5e and f, large numbers of green-stained live bacteria and only few red-stained dead bacteria were observed on PEEK, confirming its inherent lack of antibacterial activity. In sharp contrast, PEEK-*g*-PAM-NCl predominantly displayed red-stained bacteria with minimal green signals, indicating significant bacterial membrane disruption. Further quantitative analysis of the red fluorescence–labeled area (Fig. S6) showed that, in the presence of *S. aureus*, the PEEK-*g*-PAM-NCl group exhibited a red fluorescence coverage of 98.9%. Similarly, under *E. coli* conditions, the red fluorescence coverage in the PEEK-g-PAM-NCl group reached 99.7%. These live/dead staining results provide clear visual and quantitative evidence of the effective bactericidal activity of PEEK-g-PAM-NCl.

As shown in Figs. S7a and S8a, representative colony images demonstrated a time-dependent increase in bacterial growth on pristine PEEK surfaces for both *S. aureus* and *E. coli*. In contrast, markedly reduced colony formation was observed on PEEK-*g*-PAM-NCl samples with prolonged incubation time. Notably, bacterial colonies were barely detectable on PEEK-*g*-PAM-NCl at 12 h and almost completely eliminated at 24 h for both bacterial strains.

The quantitative time–kill curves (Figs. S7b and S8b) further confirmed these observations. For the PEEK group, bacterial counts increased continuously over time, reaching approximately 10^8^ CFU mL^−1^ at 24 h. In comparison, the PEEK-*g*-PAM-NCl group exhibited a distinct bactericidal trend. After a slight increase during the early incubation period, the bacterial counts began to decline significantly after 8 h. At 24 h, the bacterial viability in the PEEK-*g*-PAM-NCl group decreased by more than 0–3.7 log units relative to the PEEK group, approaching the detection limit, indicating an effective and sustained bactericidal activity.

In addition, to further simulate the antibacterial performance under in vivo–relevant conditions, antibacterial assays were conducted in a protein-rich environment. As shown in Fig. S9, after 12 h of co-culture, PEEK-*g*-PAM-NCl exhibited release rates of 93.91% against *S. aureus* and 95.94% against *E. coli*, as well as contact antibacterial rates of 91.65% and 93.97%, respectively. Furthermore, with prolonged co-culture time, as shown in Fig. S10, PEEK-*g*-PAM-NCl demonstrated markedly enhanced antibacterial performance after 24 h, achieving release antibacterial rates of 99.95% against *S. aureus* and 99.96% against *E. coli*, together with contact antibacterial rates of 99.94% and 99.95%, respectively.

### 3.4 In vivo biocompatibility

C57BL/6J mice, which are easy to handle and share similar physiological and immune features with humans, are selected to evaluate the in vivo tissue compatibility and potential biological toxicity of the implants [27]. As shown in Figs. S11a,b, a subcutaneous tissue compatibility model is established on the dorsal region of the mice. At one week post-implantation, the animals are euthanized for specimen collection and in vivo assessment. As illustrated in Fig. 5a, both PEEK and PEEK-*g*-PAM-NCl exhibit good wound healing at the implantation site, with no signs of suppuration or infection on the skin. Upon dissection, the implants are encapsulated by transparent fibrous tissue, and removal of the implants reveals intact overlying skin without necrosis or infectious manifestations. Further histological examination of the peri-implant subcutaneous tissue (Fig. 5b and c) shows only a minor infiltration of neutrophils and macrophages around both groups. Considering the inherent antibacterial activity of the N-halamine moiety, histological evaluation of major organs is also performed after one week of implantation. As shown in Fig. S12, the heart, liver, and kidney of both groups display normal anatomical structures, including intact myocardial fibers, hepatic lobules, and renal glomeruli, without any pathological abnormalities.

To further clarify the potential in vivo biotoxicity of the modified material after implantation, as shown inFig. S13a and b, an SD rat femoral bone defect model was established. Blood routine parameters were assessed at 3 days post-implantation, and peri-implant tissues were harvested for evaluation after the rats were sacrificed at 6 weeks post-implantation. As shown in Fig. S14, no statistically significant differences were observed between the PEEK and PEEK-*g*-PAM-NCl groups in terms of white blood cell, neutrophil, or monocyte counts. Furthermore, as shown in Fig. 5d and S15, radiographic examinations at 6 weeks post-implantation revealed comparable in vivo performance between the PEEK and PEEK-*g*-PAM-NCl groups, with no significant differences in newly formed bone on the implant surface or surrounding trabecular bone–related parameters. In addition, H&E staining (Fig. 5e) demonstrated the absence of abnormal neutrophil infiltration around the implants in both groups. Masson trichrome staining further showed no significant differences in peri-implant new bone formation or trabecular bone architecture between the two groups (Fig. 5f).

### 3.5 In vivo antibacterial assessment

**Fig. 6:**
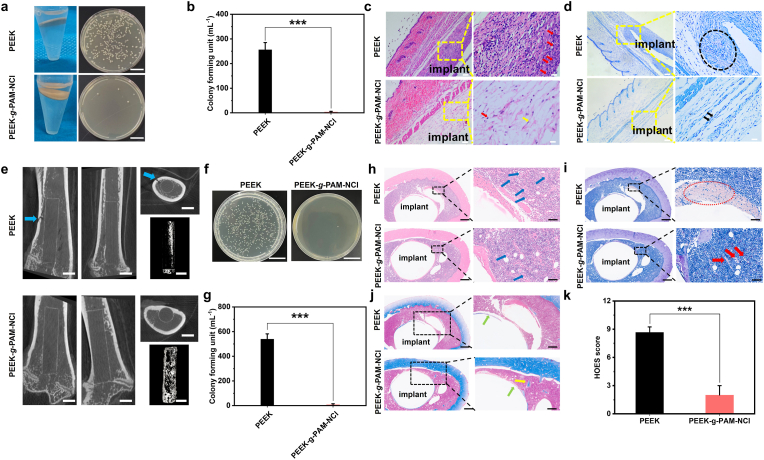
**In vivo antibacterial properties**. (a) Digital photos of bacterial colonies units from the implant surfaces in mouse's PEEK and PEEK-*g*-PAM-NCl groups (scale bars = 2 cm). (b) Quantitative analysis of in vivo bacterial colony units in the mouse's PEEK and PEEK-*g*-PAM-NCl groups. (c) H&E staining and (d) Giemsa staining results (red arrows indicate neutrophils, yellow arrows indicate fibroblasts, *S. aureus* within black circles, black arrows indicate *S. aureus*; left scale bars = 100 μm, right scale bars = 20 μm). (e) Micro-CT scanning of femurs implanted with PEEK and PEEK-*g*-PAM-NCl and the corresponding reconstructed 3D images in the rat femoral bone osteomyelitis model (the blue arrow points to the sinus tract; scale bars = 2 mm). (f) Digital photos of bacterial colonies units from the implant surfaces in rats' PEEK and PEEK-*g*-PAM-NCl groups (scale bars = 2 cm). (g) Quantitative analysis of in vivo bacterial colony units in the rats' PEEK and PEEK-*g*-PAM-NCl groups. (h) H&E staining, (i) Giemsa staining (The blue arrow points to clustered neutrophils, the red arrow and red circle represent bacteria; left scale bars = 500 μm, right scale bars = 50 μm) and (j) Masson trisome staining (yellow arrow points to the trabecular bone, and the green arrow points to the newly formed bone around the implant; left scale bars = 500 μm, right scale bars = 250 μm) of peri-implanted femur in PEEK and PEEK-*g*-PAM-NCl groups in the femoral bone osteomyelitis model of SD rats. (k) Quantitative analysis of HOES score in the rats' PEEK and PEEK-*g*-PAM-NCl groups. The data are mean ± SD. ∗*p* < 0.05, ∗ ∗*p* < 0.01, ∗ ∗ ∗*p* < 0.001, ns means not statistically significant.

To further verify the in vivo antibacterial efficacy of our modification strategy, a subcutaneous implant-associated infection model is established in C57BL/6J mice (Fig. S16a). Unlike the subcutaneous biocompatibility model, bacteria are co-implanted during model construction to mimic the clinical environment of surgical site infection (SSI), a common complication that typically occurs within two weeks post-surgery and represents the third most frequent cause of readmission following spinal surgery, with an incidence of approximately 3.1% [28,29]. At 2 weeks post-implantation, the mice are sacrificed, and to minimize interference from commensal bacteria such as hair follicle flora, only the bacterial burden on implant surfaces is qualitatively and quantitatively evaluated. As shown in Fig. 6a, PEEK-implanted samples cause turbid PBS suspensions and yield abundant *S. aureus* colonies on agar plates, whereas PEEK-*g*-PAM-NCl–implanted samples maintain clear PBS suspensions with only sparse colonies. Quantitative analysis further confirms this finding (Fig. 6b), where an average of 256 colonies is detected on PEEK compared to only 4 colonies on PEEK-g-PAM-NCl, corresponding to a 98.6% antibacterial efficacy against *S. aureus.*

Consistently, examination of peri-implant subcutaneous tissue reveals evident necrosis in the PEEK group, while normal pale-red skin tissue without signs of necrosis or purulence is observed in the PEEK-*g*-PAM-NCl group (Fig. S16b). Histological evaluation provides further validation: H&E staining demonstrates massive neutrophil infiltration in the peri-implant tissue of the PEEK group, whereas only sporadic neutrophils are observed in the PEEK-*g*-PAM-NCl group (Fig. 6c). Likewise, Giemsa staining indicates abundant purple-stained *S. aureus* clusters in the PEEK group, but substantially fewer bacteria are detected in the PEEK-*g*-PAM-NCl group (Fig. 6d).

Similarly, further evaluation was conducted using an SD rat femoral osteomyelitis model (Fig. S17). At 3 days post-implantation, as shown in Fig. S18, the WBC, NEUT, and MONO counts in the PEEK-*g*-PAM-NCl group approached normal levels, whereas the PEEK group exhibited hematological features indicative of infection. At 6 weeks post-implantation, as shown in Fig. 6e, the PEEK group presented with evident sinus tract formation, representing typical features of chronic osteomyelitis, while the PEEK-*g*-PAM-NCl group showed an overall normal femoral appearance. Quantitative analysis of peri-implant bone tissue and trabecular parameters (Fig. S19) revealed a marked reduction in surrounding bone mass and trabecular structure in the PEEK group. Furthermore, bacterial analysis on the implant surfaces (Fig. 6f) demonstrated an average of approximately 539 colonies on PEEK, compared with only 8 colonies on PEEK-*g*-PAM-NCl, corresponding to an antibacterial efficacy of approximately 98.4% against *S. aureus* (Fig. 6g). Consistently, H&E staining (Fig. 6h) showed abundant clustered neutrophil infiltration and focal necrotic regions around the implants in the PEEK group, whereas these pathological features were markedly attenuated in the PEEK-*g*-PAM-NCl group. Giemsa staining (Fig. 6i) further revealed extensive bacterial aggregation within peri-implant vacuoles in the PEEK group, while only sparse bacterial distribution was observed in the PEEK-*g*-PAM-NCl group. Further quantitative analysis (Fig. S20) indicated a nuclear density of approximately 3386 cells mm^−2^ and an inflammatory cell area ratio of approximately 53.3% in the PEEK group, compared with a nuclear density of approximately 1144 cells mm^−2^ and an inflammatory area proportion of approximately 21.6% in the PEEK-*g*-PAM-NCl group. Masson trichrome staining (Fig. 6j) demonstrated results consistent with the micro-CT findings. In addition, HOES scoring (Fig. 6k) showed a significantly higher score in the PEEK group (approximately 9 points) compared with the PEEK-*g*-PAM-NCl group (approximately 2 points).

## 4 Discussion

PEEK itself lacks intrinsic antibacterial properties. During long-term implantation, once infection occurs, bacterial colonization around the implant is inevitable, which weakens its integration with the surrounding tissue and further leads to implant loosening and eventual failure [30]. To address this critical clinical problem, numerous antibacterial modification strategies have been investigated, including metallic or metal-compound coatings [9,31,32], physical blending with inorganic compounds [33], and grafting of organic antibacterial agents [10]. However, the biological toxicity of heavy metal ions in vivo [34], the alterations of PEEK's mechanical properties (e.g., modulus and strength) induced by physical blending that compromise stability under load-bearing conditions [35], as well as the inevitable issue of antibiotic resistance associated with organic antibacterial agents (e.g., antibiotics), have collectively limited the applicability of these strategies in bioactive materials.

In this study, we propose a novel biofriendly and antibiotic-free antibacterial modification strategy for PEEK, based on its unique benzophenone-like structure. Under UV irradiation, PEEK generates semi-benzopinacol radicals, which serve as active sites to initiate graft polymerization of monomers with –C=C– double bonds (main reaction) and to form covalent bonds between the propagating radical ends of grafted polymers and the PEEK backbone (side reaction) [36]. This approach enables covalent grafting of polyacrylamide (PAM) brushes onto PEEK, followed by chlorination to convert hydrophilic N–H groups into antibacterial N–Cl functionalities, thereby endowing PEEK with antibacterial properties. As this is a surface modification strategy, the bulk structure of PEEK remains unaffected, preserving its superior mechanical strength and modulus that match those of bone. This has been validated in detail by Kyomoto et al. [37] and will not be reiterated here. Compared to non-covalent coating strategies, this covalent grafting approach provides robust and durable interfacial bonding between the polymer brushes and the PEEK substrate.

Currently, antibacterial modification strategies for biomaterial substrates can be broadly classified into two categories: physical and chemical bactericidal mechanisms [[38], [39], [40]]. Physical mechanisms include altering surface nanotopography via nanoparticles or nanoneedles, mimicking superhydrophobic surfaces such as lotus leaves or cicada wings, and introducing photothermal conversion agents such as graphene [41]. In contrast, chemical bactericidal mechanisms offer greater diversity, efficiency, broad-spectrum activity, and reduced damage to normal tissues [42,43]. Among them, N-halamines (with the general structure N–X, where X = halogen) are well known for their high efficacy and durability. Because of its high electron affinity, chlorine (Cl) is commonly used, with the N–Cl bond demonstrating excellent stability [44]. Regarding precursor selection, Oril et al. [23] reported the aqueous stability of halogenated groups in the order: amine > amide > imide. This indicates that N–amine species are highly stable but show limited antibacterial efficacy due to poor dissociation, whereas N–imide species dissociate too rapidly in water, causing a short-lived “burst release” of active chlorine and loss of long-term antibacterial protection [45]. Therefore, in this study, acrylamide was chosen as the monomer, producing N–Cl bonds that dissociate at a controlled rate while retaining un-dissociated N–Cl bonds within the polymer chain. In vivo implantation studies, including a subcutaneous implant-associated infection model in C57BL/6J mice and a femoral osteomyelitis model in SD rats, confirmed that the PEEK-*g*-PAM-NCl group maintained up to 98% antibacterial efficacy even after 14 days and 6 weeks, respectively, achieving long-lasting and stable bactericidal effects within the investigated time frame against *S. aureus*.

The bactericidal mechanism of N–Cl functionalities involves the gradual release of oxidative Cl^+^ ions, which exert strong, non-specific oxidative damage to bacterial cell membrane proteins, leading to bacterial inactivation and denaturation [46]. Additionally, free Cl^+^ participates in ionic exchange processes that disrupt bacterial metabolism, thus producing both extracellular and intracellular antibacterial effects [45]. Meanwhile, un-dissociated N–Cl bonds retain strong oxidative capacity and can directly oxidize thiols, thioethers, and other reductive membrane proteins upon contact, achieving effective contact-based bactericidal activity [47]. Unlike conventional antibiotics, PEEK-*g*-PAM-NCl exhibits broad-spectrum and potent antibacterial activity against common implant-associated pathogens (e.g., *S. aureus*, *E. coli*, and *P. aeruginosa*), fungi (*C. albicans*), and multidrug-resistant bacteria (e.g., MRSA) in vitro. In line with this oxidative antibacterial mechanism, N-halamine chemistry is generally considered less prone to inducing bacterial resistance, which may represent a potential advantage over conventional antibiotics and contributes to reduced bacterial adhesion. These results demonstrate that the proposed strategy enables the introduction of antibacterial functionality onto an otherwise biologically inert PEEK surface using only p.p.m.-level N-Cl bonds.

Interestingly, antibacterial testing against *S. aureus* (Gram-positive cocci) and *E. coli* (Gram-negative rods) revealed stronger antibacterial effects against *E. coli*. This may be attributed to differences in bacterial cell wall structure [48]: Gram-positive bacteria possess a thick peptidoglycan layer, whereas Gram-negative bacteria have thinner walls, allowing Cl^+^ ions released from N–Cl groups to penetrate more easily and disrupt metabolic processes, thereby enhancing antibacterial efficacy against Gram-negative bacteria under equivalent conditions.

As a long-term implant material, PEEK must maintain excellent biocompatibility in addition to strong and durable antibacterial activity to minimize adverse effects on normal tissues and cells [49]. In vitro and in vivo biocompatibility evaluations demonstrated that modified PEEK-g-PAM-NCl did not significantly affect MSC viability compared to controls, and subcutaneous implantation in C57BL/6J mice confirmed tissue compatibility comparable to unmodified PEEK. This can be explained by two factors: (1) polyacrylamide-based polymers are already widely applied in skin sensors [50], drug-delivery scaffolds [51], wound healing [52], tissue adhesives [53], wound dressings [54], and hemostats [55], and thus are known to possess excellent biocompatibility; (2) N-halamines are already extensively used in food packaging [56] and medical devices [57], and the ppm-level surface N–Cl content of PEEK-*g*-PAM-NCl imposes minimal impact on cells and tissues.

Reactive oxygen species (ROS) are involved in antimicrobial processes; however, the present N-halamine–functionalized system was not designed to provide antioxidant activity. DPPH assays demonstrated that PEEK-*g*-PAM-NCl exhibited negligible radical-scavenging activity, indicating that its antibacterial efficacy is not associated with ROS scavenging but rather with oxidative chlorine species and contact-mediated mechanisms.

Despite its promising performance, the strategy also presents limitations. From a materials perspective, storage stability testing revealed that under standard conditions, surface antibacterial groups showed minimal loss and could be fully restored to initial chlorine levels by rechlorination. However, under UV irradiation, N–Cl groups degraded more substantially, and rechlorination failed to fully restore chlorine levels. It should be noted that this UV-induced degradation is primarily relevant to material storage, surface processing, or sterilization procedures, rather than in vivo implantation, as PEEK implants are not exposed to ultraviolet radiation in clinical settings. Two mechanisms contribute: (1) UV-induced cleavage of N–Cl bonds produces N–H groups that can be partially re-chlorinated; (2) under UV exposure, Hofmann–Löffler rearrangement of N–Cl groups occurs, where chlorine atoms migrate to polymer side chains, resulting in permanent deactivation of binding sites and irreversible chlorine loss [58,59]. Current strategies to improve UV stability of N–Cl groups mainly involve altering N-halamine precursors (e.g., introducing epoxy groups [60] or aromatic structures [61]) or adding UV absorbers such as 2,4-dihydroxybenzophenone [62] or nano-TiO_2_ [63]. Nevertheless, these approaches only partially improve stability and cannot fully resolve the intrinsic UV instability of N–Cl groups.

Beyond material stability, translational considerations must also be addressed. From a biological and translational perspective, although the present in vivo investigations included a 6-week implantation period that enabled evaluation of subacute infection control, tissue response, and early bone remodeling, longer-term implantation studies will still be required to comprehensively assess chronic safety, antibacterial durability, and long-term bone–implant interactions. In addition, the in vivo infection models employed *S. aureus* as a single clinically relevant pathogen. While this choice reflects the most common causative organism in orthopedic implant–associated infections, future studies incorporating additional bacterial species or polymicrobial infection models would further enhance the translational relevance of the findings.

Furthermore, although quantitative histological assessments—including HOES scoring, inflammatory area proportion, and nuclear density—were performed and consistently demonstrated reduced inflammation and bacterial burden in the PEEK-*g*-PAM-NCl group, more spatially resolved and longitudinal histomorphometric analyses may provide deeper insights into the progression and heterogeneity of infection-related tissue responses. These aspects will be systematically explored in future investigations.

## 5 Conclusion

In conclusion, this study exploits the intrinsic structural features of PEEK to achieve antibacterial functionalization through a simple and quasi-nondestructive surface modification strategy. Without compromising biocompatibility, UV-induced in situ self-initiation and subsequent halogenation enable the introduction of N-halamine functionalities onto PEEK surfaces. Both in vitro and in vivo evaluations demonstrate broad-spectrum antimicrobial activity while maintaining favorable biocompatibility, indicating its potential for reducing the risk of implant-associated infections.

## Ethics approval statement

This study was approved by the Ethics Committee of the Xi'an Honghui Hospital of Xi'an Jiaotong University. Animal experiments on C57BL/6J mice subcutaneous tissue compatibility models, subcutaneous tissue implant-associate infection models (B202305-1) and Animal experiments on SD rat femoral bone defect models, femoral osteomyelitis models (B202511-1) were approved by the Animal Ethics and Welfare Committee of Guangzhou Huateng Biomedical Technology Co., Ltd. All experimental procedures were complied with the Laboratory Animal Care and Use Guidelines in strict.

## Declaration of generative AI in scientific writing

During the preparation of this work, the authors used ChatGPT (OpenAI) in order to assist with language editing and grammatical refinement. After using this tool, the authors reviewed and edited the content as needed and take full responsibility for the content of the published article.

## Funding

“The authors acknowledge the financial support from the Natural Science Basic Resaerch Program of Shaanxi Province (Grant No.2025JC-YBQN-1269).”

## Conflict of interest

The authors declare no conflict of interest.
