# Supplementary material for: Antibiotic-free antimicrobial functionalization of PEEK via UV-induced self-initiation and N-halamine grafting
Source: J Orthop Translat. 2026 Mar 19;58:101081. doi: 10.1016/j.jot.2026.101081 (PMC13019069; doi:10.1016/j.jot.2026.101081)
Supplement: Multimedia component 1 [file mmc1.docx]

# **Antibiotic-Free Antimicrobial Functionalization of PEEK via UV-Induced Self-Initiation and N-Halamine Grafting**


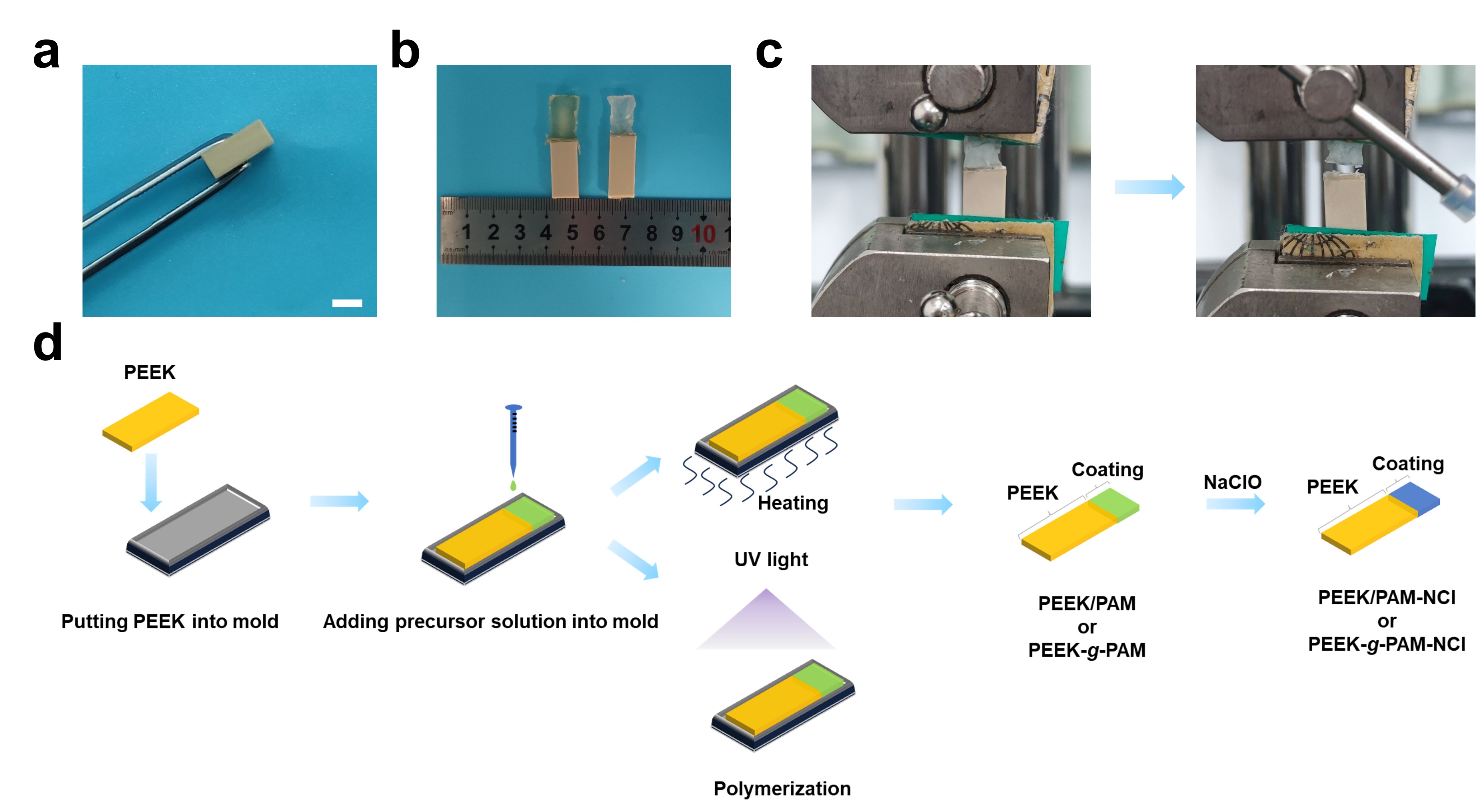


**Figure S1.** (a) Digital photo of PEEK cross-section using the pull-off test to measure bonding strength (scale bar = 4 mm). (b) Digital photo of PEEK samples after grafting the corresponding coating. (c) The process of measuring bonding strength using the pull-out method. (d) Schematic illustration of PEEK/PAM-NCl and PEEK-*g*-PAM-NCl sample preparation process.


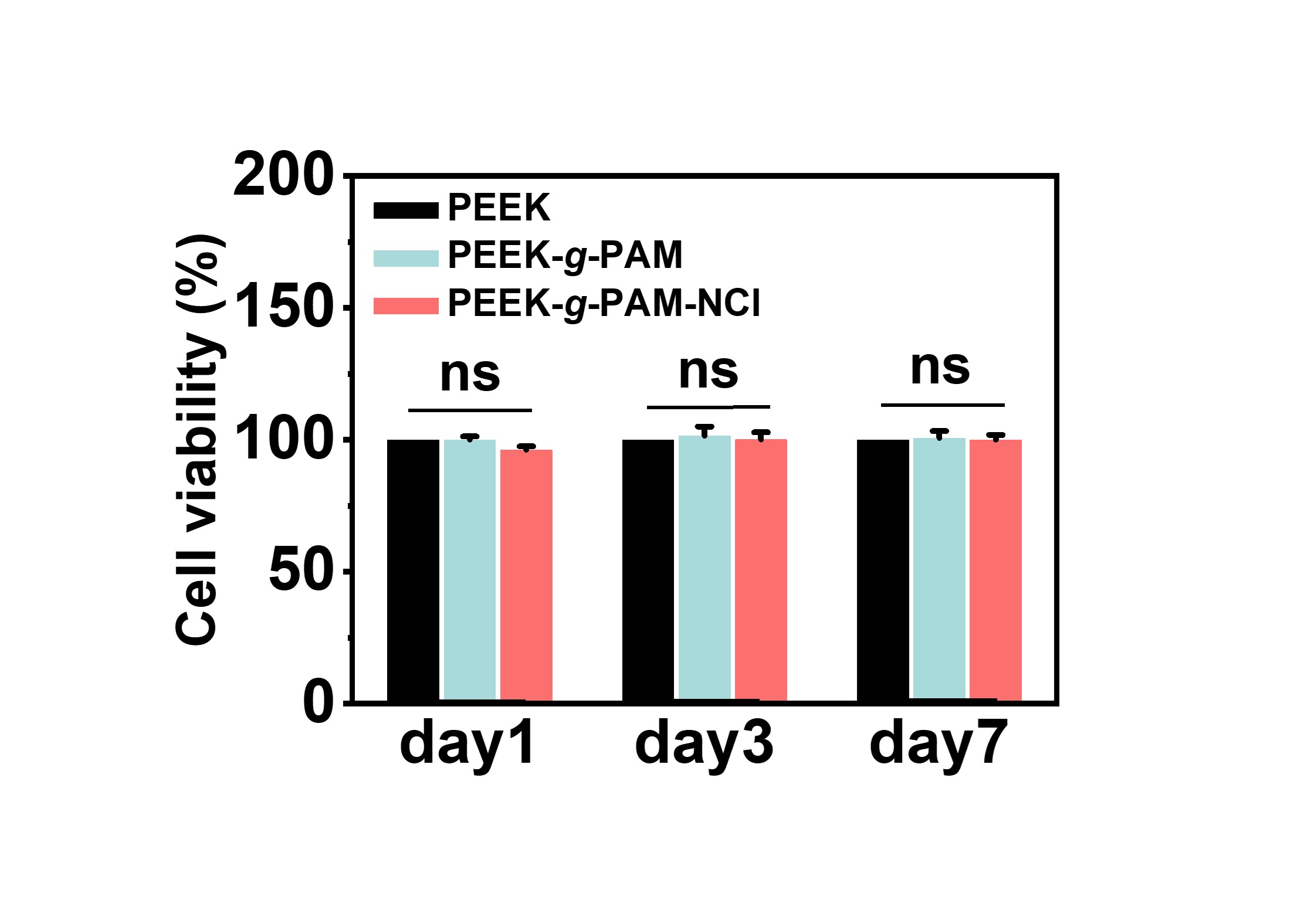


**Figure S2.** cell viability results of hBM-MSCs co-cultured with PEEK, PEEK-*g*-PAM, and PEEK-*g*-PAM-NCl for 1, 3, and 7 days.


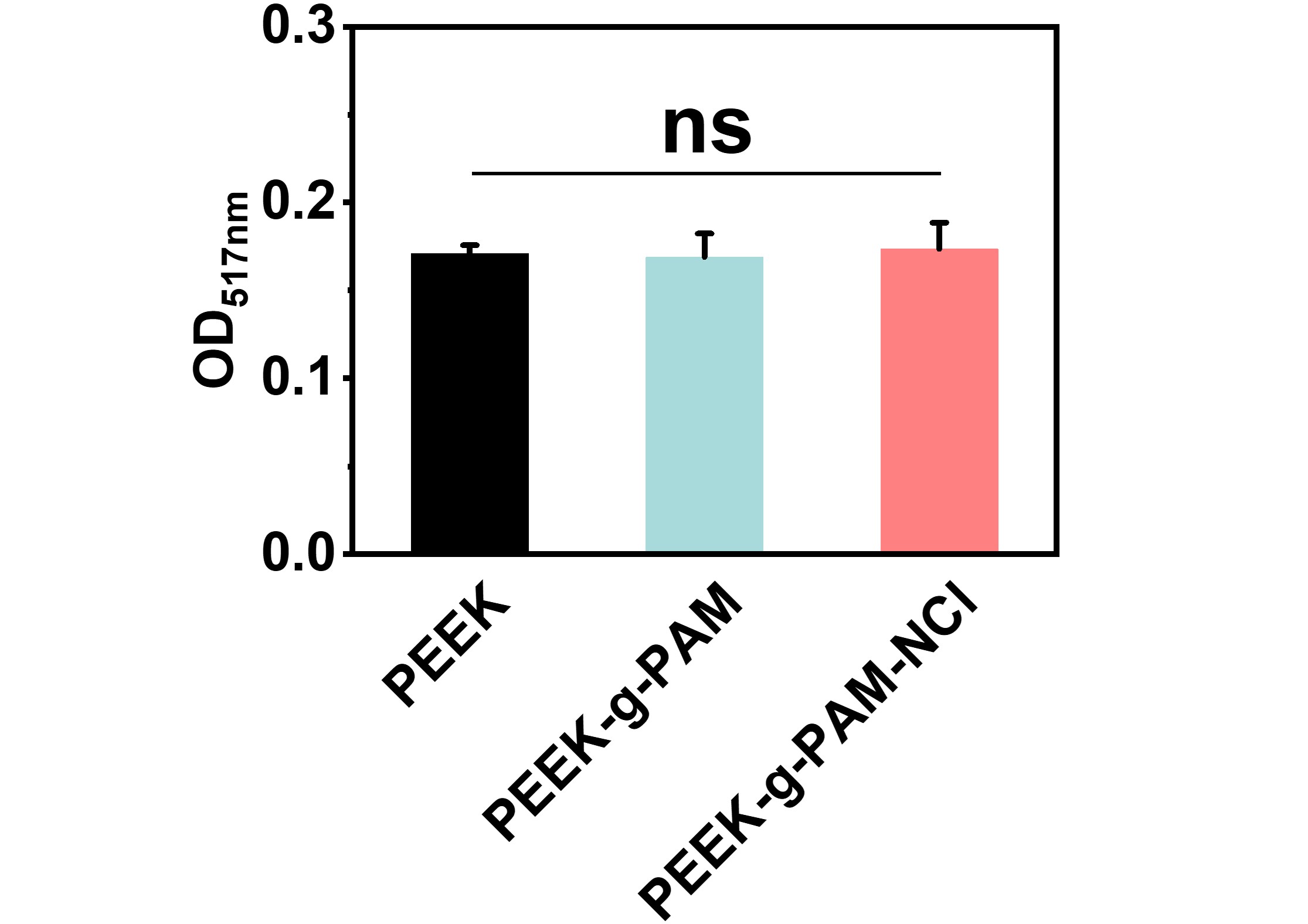


**Figure S3.** OD values of in vitro ROS scavenging efficiency of PEEK, PEEK-*g*-PAM, and PEEK-*g*-PAM-NCl.


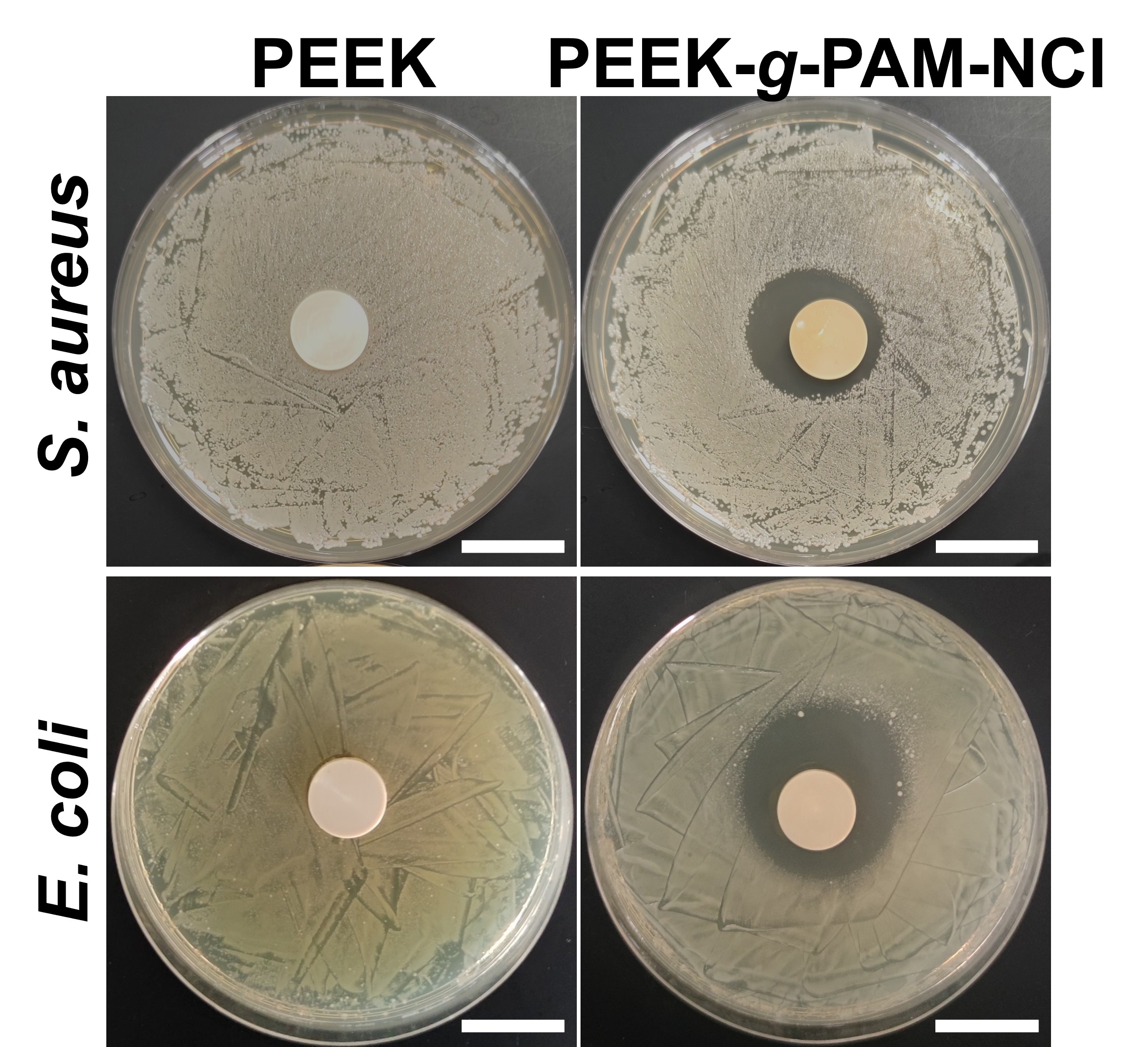


**Figure S4.** Results of the antibacterial ring assay for PEEK and PEEK-*g*-PAM-NCl (scale bar = 2 cm).


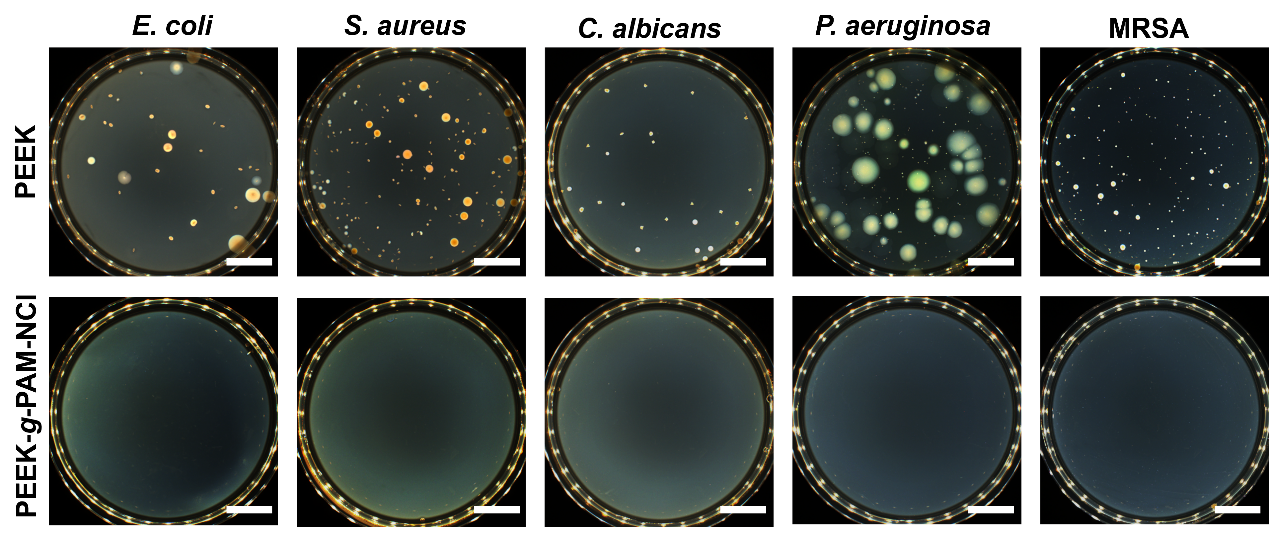


**Figure S5.** Colony images from the broad-spectrum antimicrobial test of PEEK and PEEK-*g*-PAM-NCl groups (scale bars = 2 cm).

**TableS1.** Broad-spectrum antimicrobial test results for PEEK and PEEK-*g*-PAM-NCl groups.

| **Species of microbes** | **Initial concentration^a^ (CFU mL^-1^)** | **PEEK^b^ (CFU mL^-1^)** | **PEEK-*g*-PAM-NCl^c^ (CFU mL^-1^)** | **Antimicrobial rate (%)** |
| --- | --- | --- | --- | --- |
| ***E. coli*** | 2.1×10^4^ | 4.3×10^6^ | ＜1 | ＞99 |
| ***S. aureus*** | 2.1×10^4^ | 1.7×10^5^ | ＜1 | ＞99 |
| ***C. albicans*** | 1.7×10^4^ | 3.5×10^4^ | ＜1 | ＞99 |
| ***P. aeruginosa*** | 2.4×10^4^ | 2.5×10^4^ | ＜1 | ＞99 |
| **MRSA** | 2.1×10^4^ | 1.8×10^5^ | ＜1 | ＞99 |

**Note:** **^a^**, average concentration of viable microorganisms at 0 h; **^b^**, average concentration of viable microorganisms in the PEEK group after 18 h of incubation; **^c^**, average concentration of viable microorganisms in the PEEK-g-PAM-NCl group after 18 h of incubation.


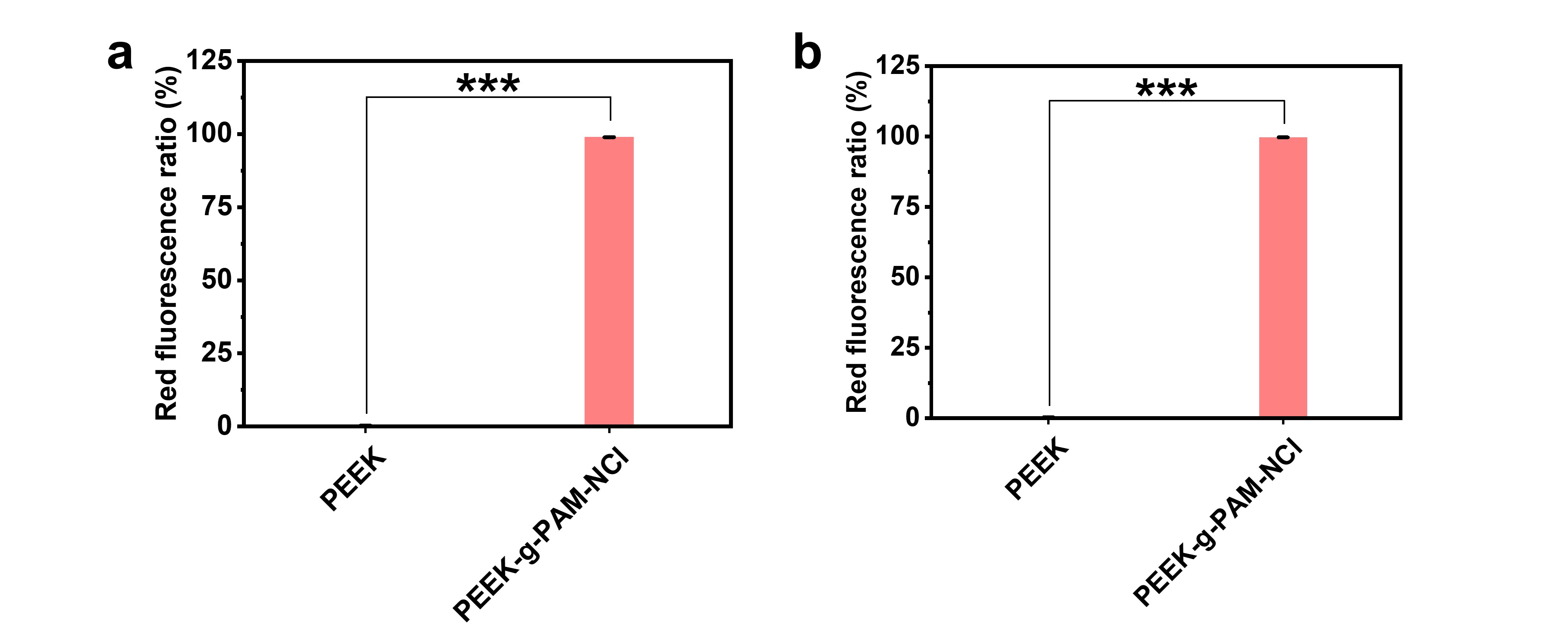


**Figure S6.** Quantitative analysis of the proportion of red fluorescent regions in *S. aureus* (a) and *E. coli* (b). The data are mean ± SD, * p<0.05, * * p<0.01, * * * p<0.001, ns means not statistically significant.


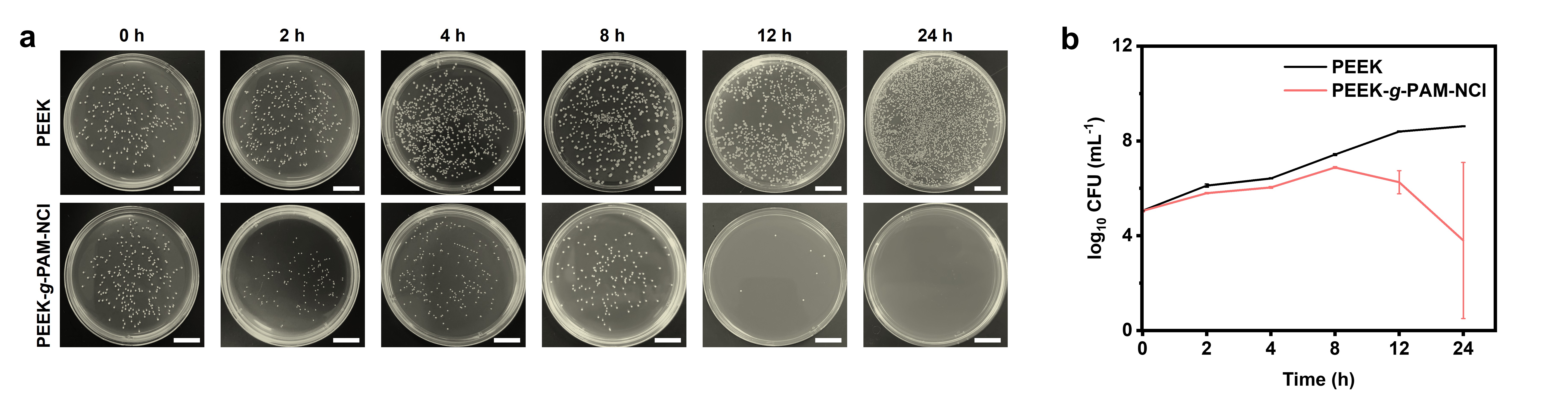


**Figure S7.** (a) Images of bacterial colony-forming units (obtained by diluting the bacterial solution 10 times, 100 times, 100 times, 1000 times, 10000 times, and 10000 times at co-culture time points, respectively; scale bars = 2 cm) and (b) corresponding bacterial killing-time curves by *S. aureus* in PEEK and PEEK-*g*-PAM-NCl samples at different time points. The data are mean ± SD, * p<0.05, * * p<0.01, * * * p<0.001, ns means not statistically significant.


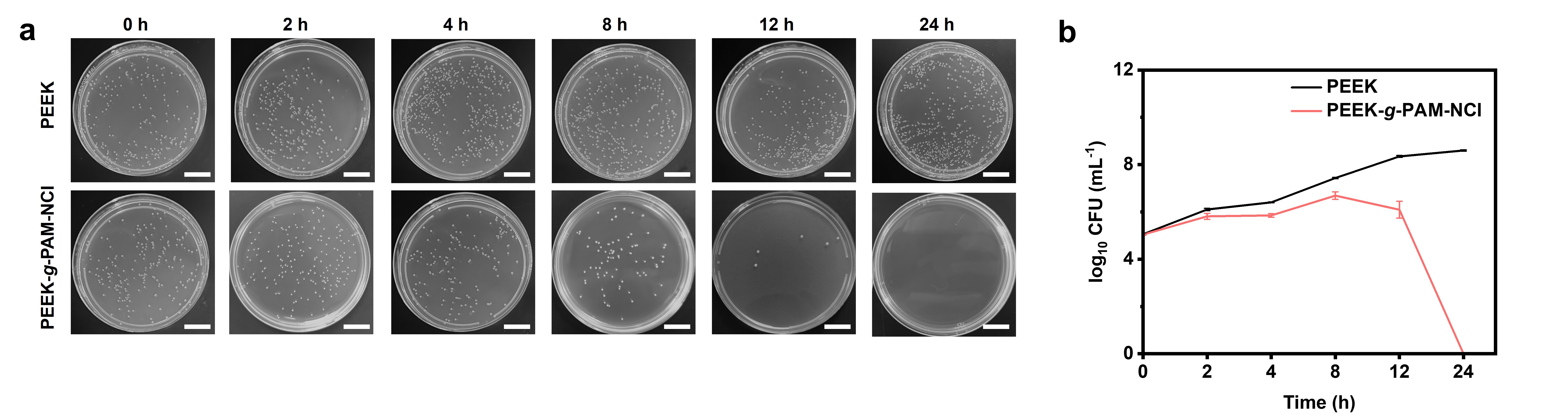


**Figure S8.** (a) Images of bacterial colony-forming units (obtained by diluting the bacterial solution 10 times, 100 times, 100 times, 1000 times, 10000 times, and 10000 times at co-culture time points, respectively; scale bars = 2 cm) and (b) corresponding bacterial killing-time curves by *E. coli* in PEEK and PEEK-*g*-PAM-NCl samples at different time points. The data are mean ± SD, * p<0.05, * * p<0.01, * * * p<0.001, ns means not statistically significant.


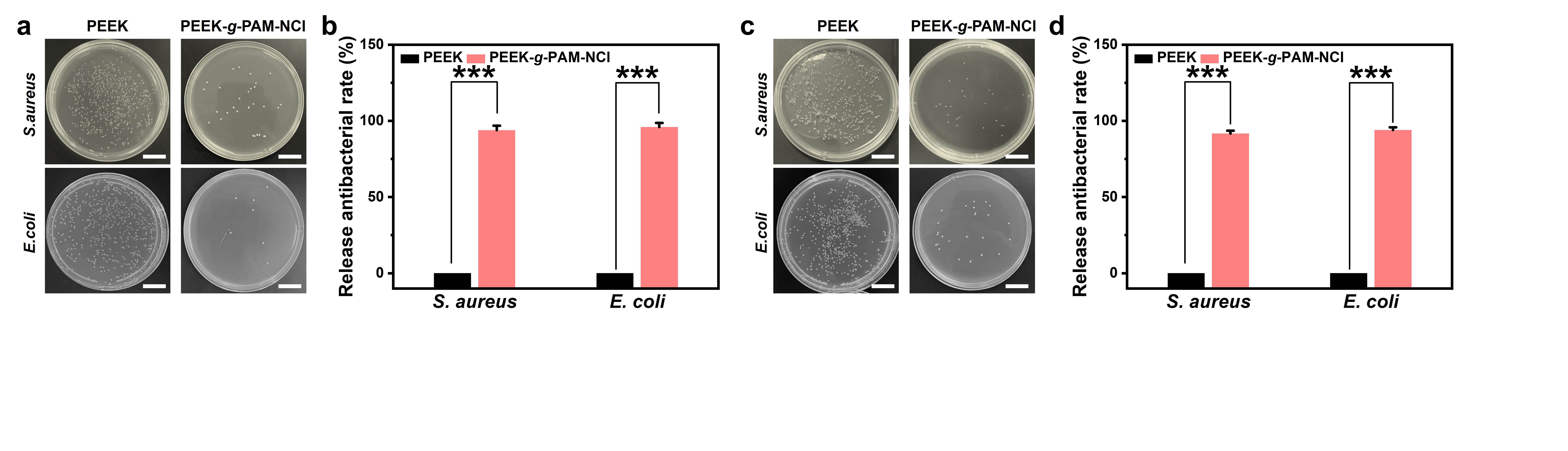


**Figure S9.** (a) Images of bacterial colony-forming units by *S. aureus* and *E. coli* in the supernatants of PEEK and PEEK-*g*-PAM-NCl under bacterial solution containing 10% FBS for 12 h (scale bars = 2 mm). (b) Release antibacterial rates against *S. aureus* and *E. coli* of PEEK and PEEK-*g*-PAM-NCl. (c) Images of bacterial colony-forming units by *S. aureus* and *E. coli* grown on PEEK and PEEK-*g*-PAM-NCl under bacterial solution containing 10% FBS for 12 h (scale bars = 2 mm). (d) Contact antibacterial rates against *S. aureus* and *E. coli* of PEEK and PEEK-*g*-PAM-NCl. The data are mean ± SD, * *p*<0.05, * * *p*<0.01, * * * *p*<0.001, ns means not statistically significant.


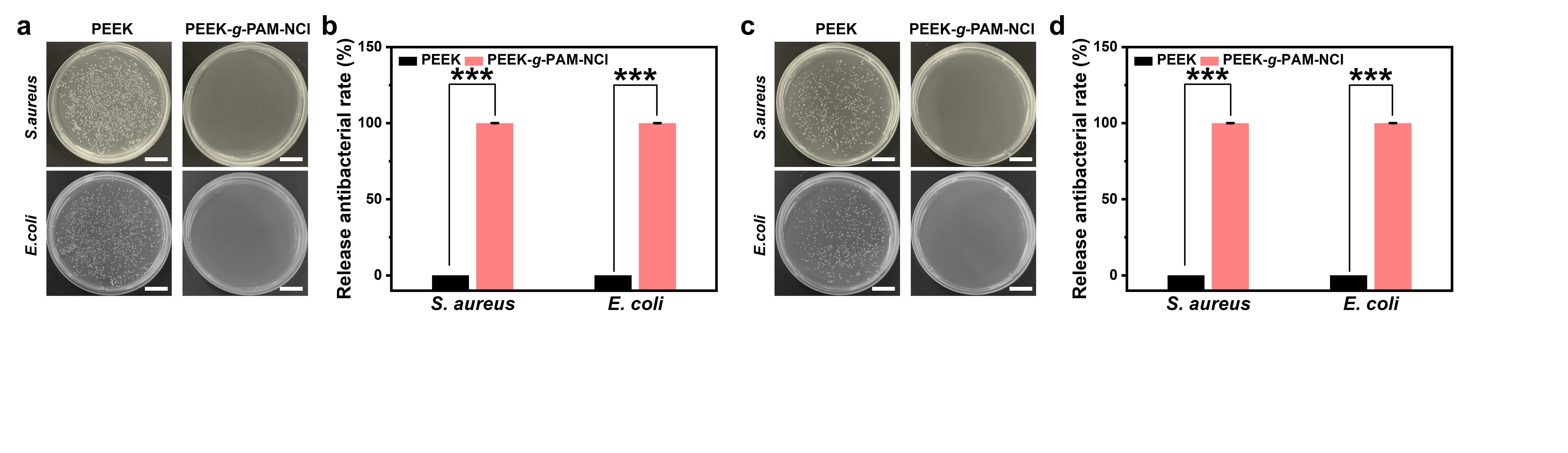


**Figure S10.** (a) Images of bacterial colony-forming units by *S. aureus* and *E. coli* in the supernatants of PEEK and PEEK-*g*-PAM-NCl under bacterial solution containing 10% FBS for 24 h (scale bars = 2 mm). (b) Release antibacterial rates against *S. aureus* and *E. coli* of PEEK and PEEK-*g*-PAM-NCl. (c) Images of bacterial colony-forming units by *S. aureus* and *E. coli* grown on PEEK and PEEK-*g*-PAM-NCl under bacterial solution containing 10% FBS for 24 h (scale bars = 2 mm). (d) Contact antibacterial rates against *S. aureus* and *E. coli* of PEEK and PEEK-*g*-PAM-NCl. The data are mean ± SD, * *p*<0.05, * * *p*<0.01, * * * *p*<0.001, ns means not statistically significant.


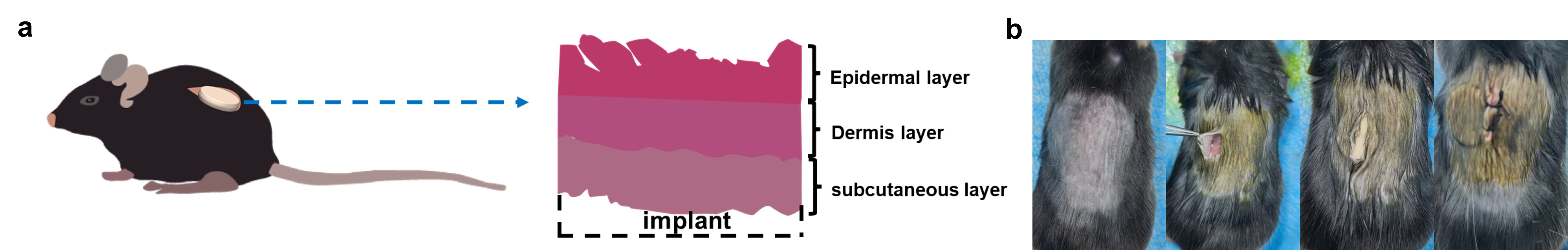


**Figure S11.** Schematic diagram (a) and (b) digital photos of subcutaneous tissue compatibility model in the C57BL/6J mice.


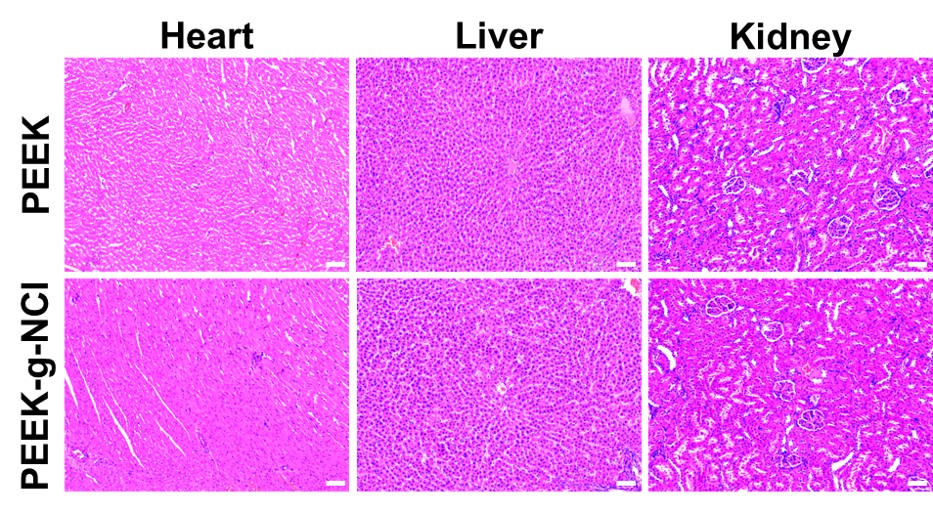


**Figure S12.** H&E staining results of major organs (heart, liver, kidney) 1 week after implantation of PEEK and PEEK-*g*-PAM-NCl in C57BL/6J mice (scale bars = 20 µm).


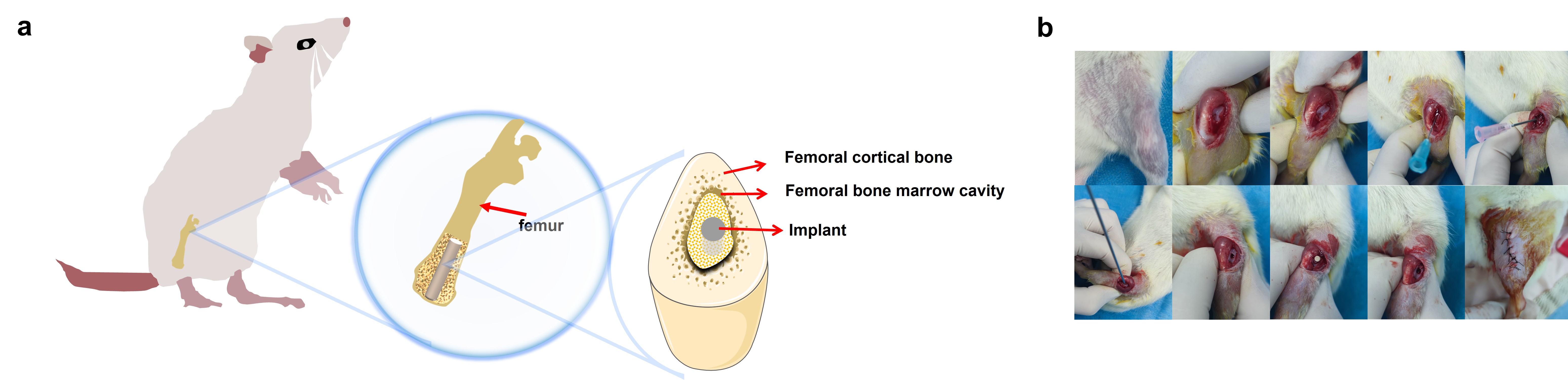


**Figure S13.** (a) Schematic illustration and (b) digital photos of femoral bone defect modeling in SD rats.


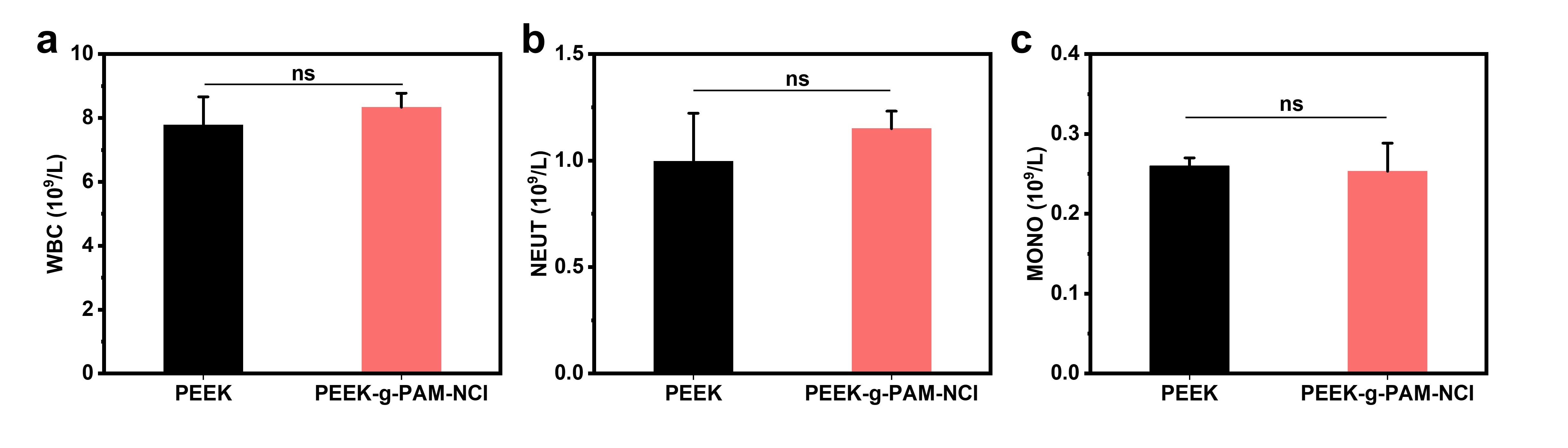


**Figure S14.** Levels of (a) white blood cells (WBC), (b) neutrophils (NEUT) and (c) monocytes (MONO) after the implantation of femoral bone defect modeling in SD rats for 3 days (n=3). The data are mean ± SD, * *p*<0.05, * * *p*<0.01, * * * *p*<0.001, ns means not statistically significant.BV


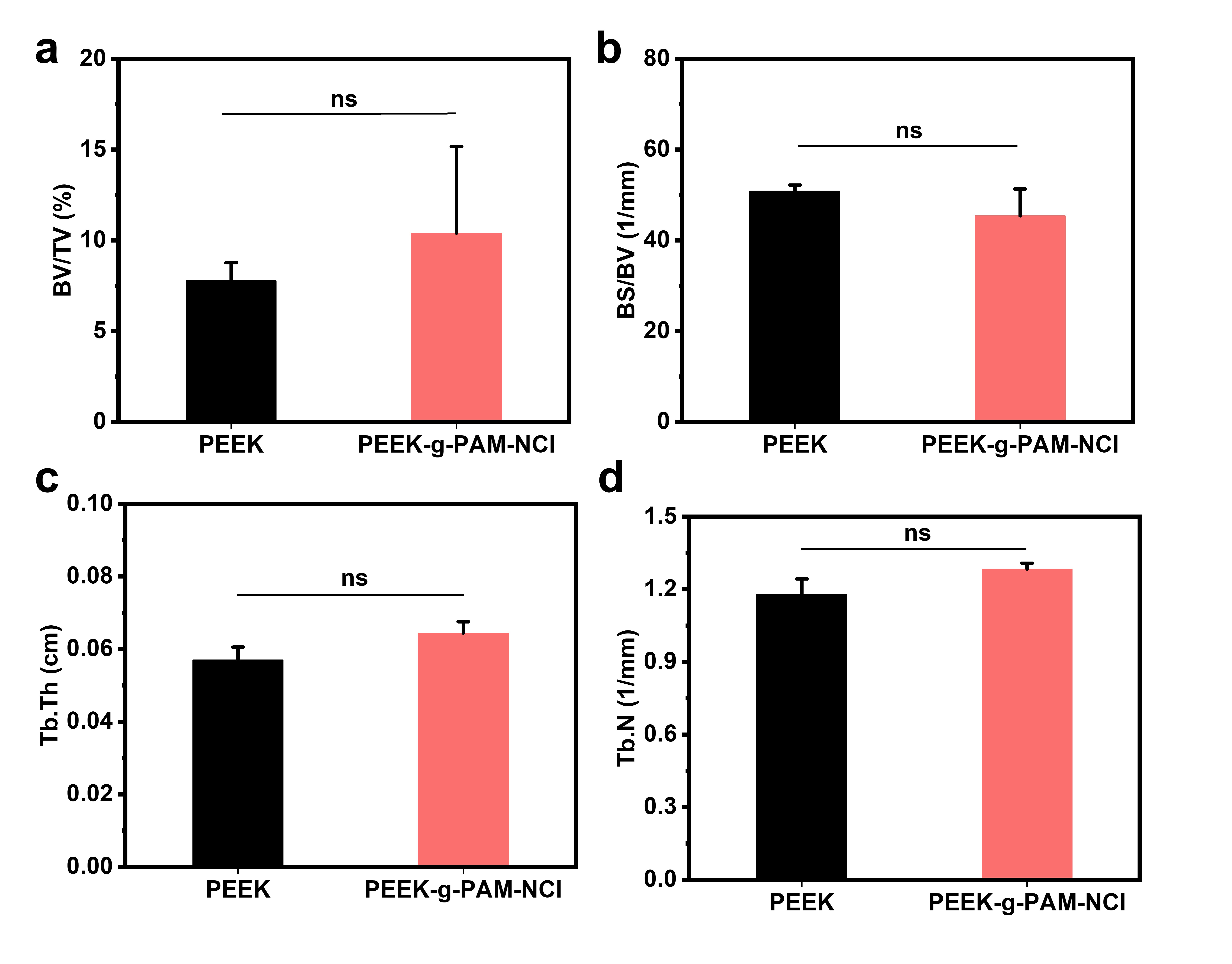


**Figure S15.** The quantitative statistical results of (a) BV/TV, (b) BS/BV, (c) Tb.Th and (d) Tb.N of femoral bone defect modeling in SD rats for the PEEK and PEEK-*g*-PAM-NCl groups.


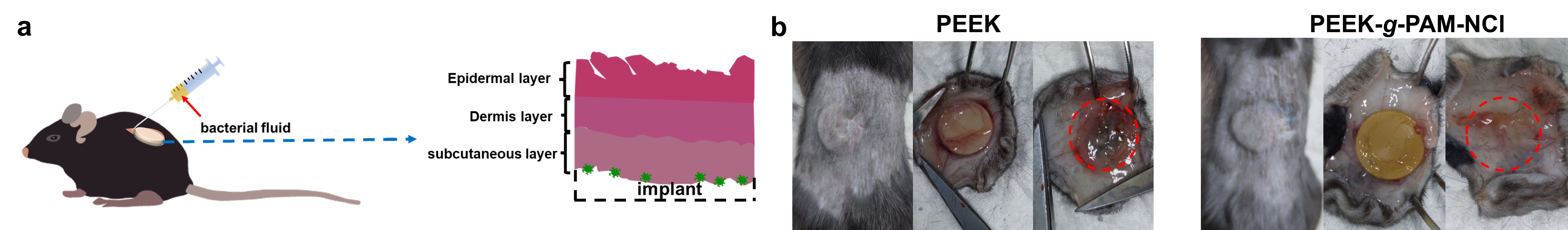


**Figure S16.** (a) Schematic diagram of the subcutaneous implant-associated infection model in the C57BL/6J mice. (b) Digital photos of the dorsal skin, tissues surrounding the implants, and dorsal skin after removal of implants in PEEK and PEEK-*g*-PAM-NCl groups, 2 weeks after implantation in the subcutaneous implant-associated infection model in C57BL/6J mice.


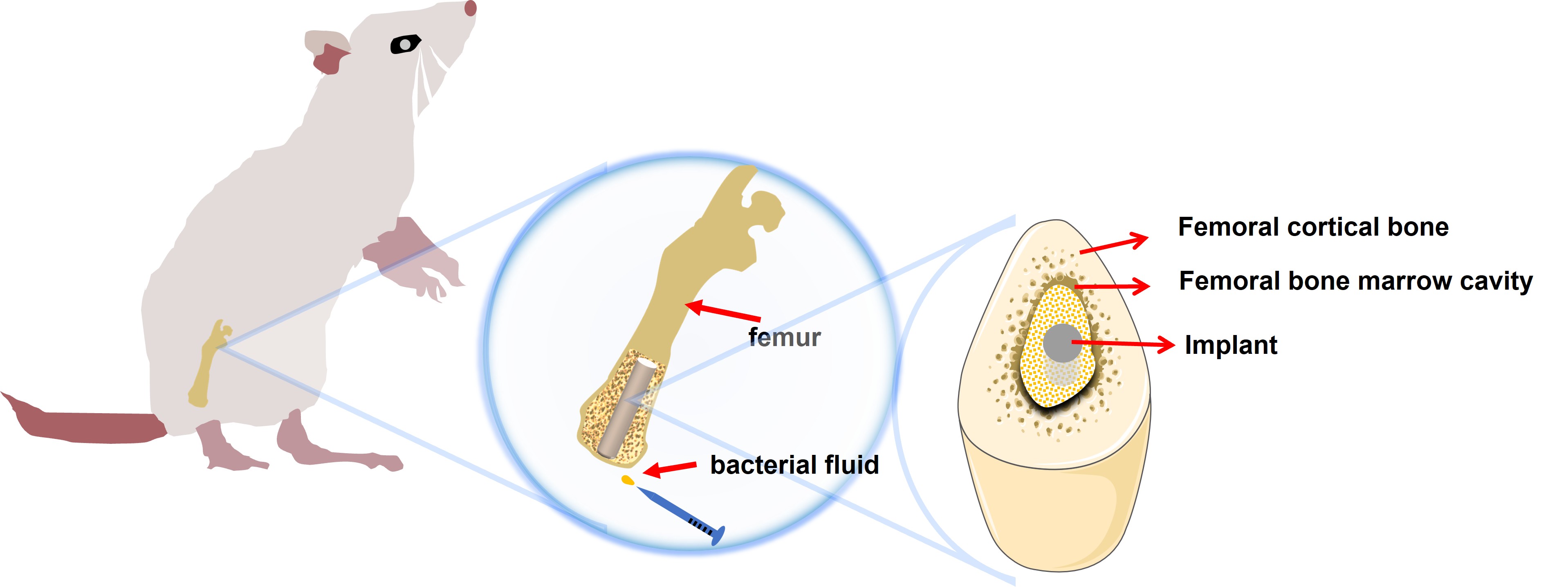


**Figure S17.** Schematic diagram of the femoral osteomyelitis model in SD rat.


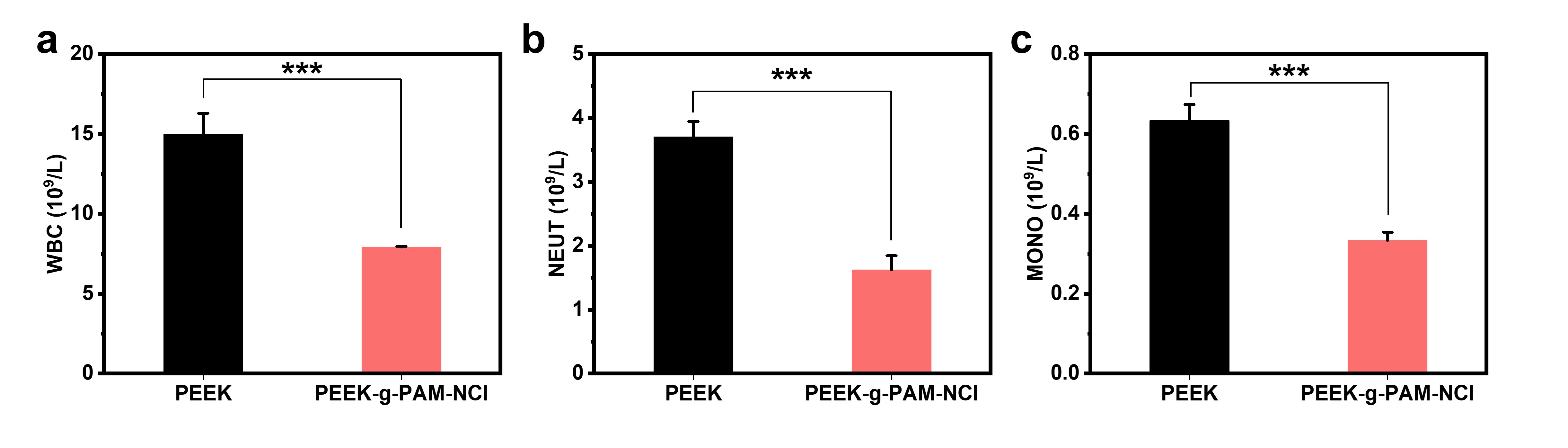


**Figure S18.** Levels of (a) white blood cells (WBC), (b) neutrophils (NEUT) and (c) monocytes (MONO) after the implantation of femoral bone osteomyelitis model in SD rat for 3 days (n=3). The data are mean ± SD, * *p*<0.05, * * *p*<0.01, * * * *p*<0.001, ns means not statistically significant.


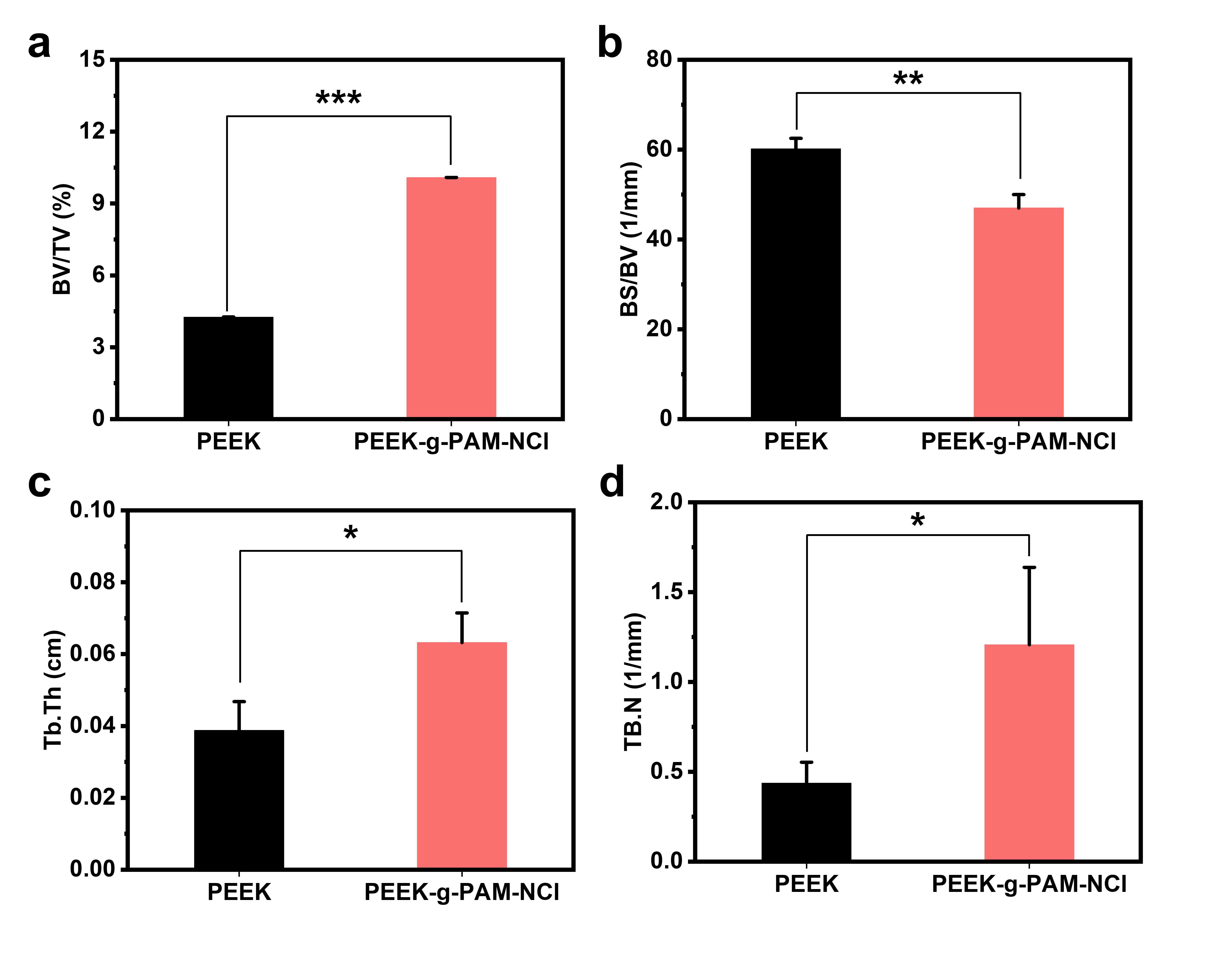


**Figure S19.** The quantitative statistical results of (a) BV/TV, (b) BS/BV, (c) Tb.Th and (d) Tb.N of femoral bone osteomyelitis model in SD rats for the PEEK and PEEK-*g*-PAM-NCl groups.


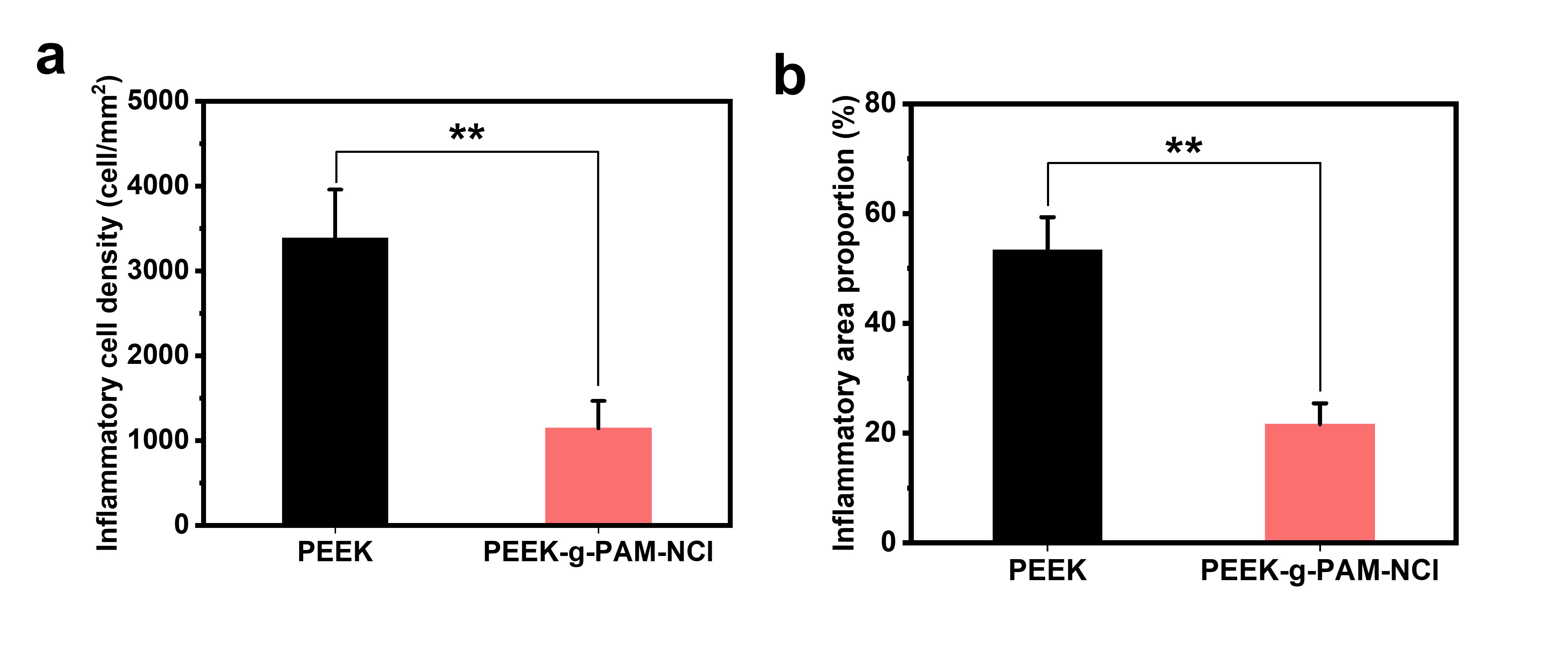


**Figure S20.** Quantitative analysis of the inflammatory cell density (a) and inflammatory area proportion (b).
